# Promiscuous Binding of Karyopherin*β*1 Modulates FG Nucleoporin Barrier Function and Expedites NTF2 Transport Kinetics

**DOI:** 10.1016/j.bpj.2014.12.041

**Published:** 2015-02-17

**Authors:** Raphael S. Wagner, Larisa E. Kapinos, Neil J. Marshall, Murray Stewart, Roderick Y.H. Lim

**Affiliations:** 1 Biozentrum and the Swiss Nanoscience Institute, University of Basel, Basel, Switzerland; 2 MRC Laboratory of Molecular Biology, Cambridge, UK

## Abstract

The transport channel of nuclear pore complexes (NPCs) contains a high density of intrinsically disordered proteins that are rich in phenylalanine-glycine (FG)-repeat motifs (FG Nups). The FG Nups interact promiscuously with various nuclear transport receptors (NTRs), such as karyopherins (Kaps), that mediate the trafficking of nucleocytoplasmic cargoes while also generating a selectively permeable barrier against other macromolecules. Although the binding of NTRs to FG Nups increases molecular crowding in the NPC transport channel, it is unclear how this impacts FG Nup barrier function or the movement of other molecules, such as the Ran importer NTF2. Here, we use surface plasmon resonance to evaluate FG Nup conformation, binding equilibria, and interaction kinetics associated with the multivalent binding of NTF2 and karyopherin*β*1 (Kap*β*1) to Nsp1p molecular brushes. NTF2 and Kap*β*1 show different long- and short-lived binding characteristics that emerge from varying degrees of molecular retention and FG repeat binding avidity within the Nsp1p brush. Physiological concentrations of NTF2 produce a collapse of Nsp1p brushes, whereas Kap*β*1 binding generates brush extension. However, the presence of prebound Kap*β*1 inhibits Nsp1p brush collapse during NTF2 binding, which is dominated by weak, short-lived interactions that derive from steric hindrance and diminished avidity with Nsp1p. This suggests that binding promiscuity confers kinetic advantages to NTF2 by expediting its facilitated diffusion and reinforces the proposal that Kap*β*1 contributes to the integral barrier function of the NPC.

## Introduction

Nuclear pore complexes (NPCs) (1) are intracellular transport hubs that mediate the rapid bidirectional traffic of hundreds of proteins, ribonucleoproteins, and metabolites across the nuclear envelope (2). Each NPC contains a 50-nm-diameter central channel (3) through which only molecules smaller than ∼40 kDa (4) or ∼5 nm in size (5) can diffuse passively (6). The movement of larger molecules is impaired by a permeability barrier generated by ∼200 intrinsically disordered phenylalanine-glycine (FG)-rich nucleoporins (FG Nups) that are tethered to the NPC transport channel surface. Although the precise mechanism by which the barrier is generated in vivo has not been resolved, in vitro the FG Nups collectively resemble molecular brushes (7,8), supramolecular hydrogel meshworks (9–11), or both (12).

The translocation of selective cargoes through NPCs is mediated by a range of soluble nuclear transport receptors (NTRs) (13). These include members of the karyopherin family (Kaps) (14), such as the 97 kDa import receptor karyopherin*β*1 (Kap*β*1 or importin*β*) (15), which recognizes specific cargoes either directly or via an adaptor Kap*α*. Kap*β*1 contains several FG repeat binding pockets that exert multivalent binding interactions with the FG Nups (15–17). Multivalency (18) leads to an enhanced binding affinity through avidity (19). In vivo, each NPC contains as many as 100 Kap*β*1 molecules at steady state (20) as a result of Kap*β*1 binding to multiple FG Nups, and this would increase molecular crowding substantially. Moreover, Kap*β*1 binding has been demonstrated to alter the conformation of four different human FG Nups (Nup214, Nup62, Nup98, and Nup153) in vitro (21,22). Such conformational behavior is nonmonotonic (i.e., nonlinear) and depends on Kap*β*1 concentration, such that FG Nup brushes collapse at low nM Kap*β*1 concentrations (7) and re-extend at higher *μ*M physiological Kap*β*1 concentrations (21,22). As a result, Kap*β*1 occupancy within the FG Nups attenuates the binding avidity of incoming Kap*β*1 molecules and expedites their dissociation kinetics by reducing the number of available FG repeats (21,22). This is evident in NPC-inspired biomimetic systems (23) and provides a plausible explanation for the dependence of transport efficiency on Kap concentration in permeabilized cell assays (24).

How the binding of Kap*β*1 to FG Nups impacts NPC barrier function and influences the binding of other NTRs to FG Nups remains poorly understood. Indeed, such binding promiscuity extends beyond the FG Nups and more generally is relevant to how intrinsically disordered proteins can bind multiple partners simultaneously (25). Here, we apply surface plasmon resonance (SPR) to investigate the effect of binding promiscuity by measuring the multivalent interaction kinetics (26), equilibrium avidities, and in situ associated conformational changes that occur in Nsp1p when nuclear transport factor 2 (NTF2) and Kap*β*1 are bound, both separately and together. NTF2 is an essential homodimeric 30 kDa transport receptor that imports the GTPase Ran from the cytoplasm into the nucleus (27). Although both NTRs exhibit avidities that vary depending on their occupancy within Nsp1p, our data show a size-dependent effect that differentiates NTF2 (small) from Kap*β*1 (large). Whereas increasing Kap*β*1 from low to physiological concentrations drove the Nsp1p brush from collapse to re-extension, NTF2 caused only collapse. As a control, brush collapse was not seen with the W7A-NTF2 mutant (28), in which the avidity for FG Nups is impaired. Finally, during promiscuous binding of NTF2 in the presence of Kap*β*1, we found that Kap*β*1 retention within Nsp1p was long-lived and prevented brush collapse when NTF2 bound. This promoted faster NTF2 dissociation kinetics and supports the proposal (21,22) that Kap*β*1 contributes together with FG Nups to generate the NPC barrier function. Thus, the amount of bound Kap*β*1 could potentially influence both NPC permeability and rapid selective transport.

## Materials and Methods

### Cloning and expression of recombinant proteins

#### Wild-type NTF2

The full-length wild-type rat NTF2 coding sequence (29) was cloned into the *NdeI* and *XhoI* sites of the T7 expression vector pET15b (Novagen), with the addition of an N-terminal His_6_-tag. The construct was transformed into *Escherichia coli* strain BL21(DE3) CodonPlus RIL, expressed, and purified using NiNTA agarose and gel filtration (Superdex S-75; GE Healthcare) as previously described (29).

#### W7A-NTF2

PCR-based, site-specific mutagenesis was used to obtain the rat W7A mutant of NTF2 as previously described (30,31). The sequence was cloned into the T7 expression vector pET15b, expressed in *E. coli* BL21(DE3), and purified using ion-exchange chromatography and gel filtration as previously described (29).

#### Nsp1p-5FF and Nsp1p-12FF

Two yeast Nsp1p FG-fragments, Nsp1p-5FF (residues 262–359; 1× FG, 4× FSFG) and Nsp1p-12FF (residues 262–492; 1× FG, 11× FSFG), were cloned via *NcoI* and *HindIII* sites into a modified pET30a vector (Novagen) whose thrombin protease recognition site was changed for TEV protease and Cys-Cys-Trp was added after its initiator Met codon. The additional Cys residues facilitated coupling to the gold SPR sensor surface, whereas the Trp residue enabled us to determine the protein concentration by measuring the optical density at 280 nm. To express proteins in BL21(DE3) CodonPlus RIL, cells were grown at 37°C in 2× TY media to OD_600_ 0.6 and induced with 1 mM isopropyl *β*-D-1-thiogalactopyranoside overnight at 25°C. The cells were lysed in 50 mM Tris-HCl pH 8.0/1 mM EGTA/25% (w/v) sucrose/8 M urea by using an EmulsiFlex C3 homogenizer (Avestin) at a pressure of 15,000 psi in the presence of 1 mM PMSF. Proteins were purified under native conditions using NiNTA agarose (Qiagen) according to the manufacturer’s instructions, and then by size-exclusion chromatography on a Superdex S-75 26/60pg column (GE Healthcare) in 20 mM Tris-HCl pH 8.0/1 mM dithiothreitol/50 mM NaCl.

#### Kap*β*1

Full-length human Kap*β*1 was cloned, expressed, and purified as previously described (21). The functionality of these proteins is conserved across species (32).

Protein quality (see Fig. S1 in the Supporting Material) was assessed by SDS-PAGE and concentrations were measured by absorption at 280 nm. Protein extinction coefficients were obtained using the ProtParam program (http://web.expasy.org/protparam/).

### SPR measurements

A four-flow cell Biacore instrument (T100; GE Healthcare) was used to measure SPR at 25°C in PBS, pH 7.2 (GIBCO by Life Technologies), as previously detailed (22). Briefly, each experiment included two reference cells and two sample cells. Reference cells were prepared by covalently grafting C_17_H_36_O_4_S (hydroxyl-terminated tri(ethylene glycol) undecane thiol, HS-(CH_2_)-(OCH_2_CH_2_)_3_-OH; Nanoscience) onto a gold sensor surface via thiol binding. Sample cells were prepared by covalently grafting cysteine-modified Nsp1p fragments onto each respective gold sensor surface followed by C_17_H_36_O_4_S to further passivate any exposed gold. Different grafting distances were obtained by changing the incubation time for the Nsp1p fragments. A 1% (w/v) bovine serum albumin (BSA; Sigma-Aldrich) solution was prepared in PBS (pH 7.2). Before experiments were conducted, Kap*β*1, NTF2, W7A-NTF2, and both Nsp1p fragments were dialyzed into PBS buffer (pH 7.2). All protein and reagent solutions were centrifuged for 15 min at 16,000 × *g* to remove particles and gas bubbles. Buffer solutions were filtered (0.22 *μ*m) and degassed before use. Postexperiment checks ensured that covalent binding of Kaps to the underlying gold surface did not occur (Fig. S2). In all cases, layer height was measured after a dissociation phase of 480 s due to technical limitations that prevented the simultaneous injection of BSA with the respective NTR. Therefore, the BSA signal obtained for the bound material *R*_bound,*i*_ underestimated the height at equilibrium binding *R*_eq,*i*_ (Fig. S3). The total number of experiments, *N*, was as follows: Kap*β*1 on Nsp1p-12FF (*N* = 8), NTF2 on Nsp1p-5FF (*N* = 11), NTF2 on Nsp1p-12FF (*N* = 15), and NTF2/Kap*β*1 on Nsp1p-12FF (*N* = 5).

### Multivalent binding analysis

A model that calculates a discrete distribution of kinetic states (*k*_on*,i*_,*k*_off*,i*_) (26) was used to fit the measured SPR sensorgrams for Kap*β*1 as previously described (21). For NTF2, we used a simplified two-dimensional lattice of 5 × 5 nm^2^ NTF2-binding spots to describe the FG-repeat-containing surface, taking the average Stokes radius of an NTF2-dimer as 2.5 nm (2) (Supporting Material and Fig. S4). In brief, a set of 36 × 36 (*k*_on*,i*_, *k*_off*,i*_) pairs was populated and their fractional abundance was depicted as color intensity in *k*_on_-versus-KD and *k*_off_-versus-KD interaction maps averaged over ∼10 individual sensorgrams. Calculations and visualizations were obtained using MATLAB (The MathWorks, Natick, MA) and Python.

## Results

### Close-packed Nsp1p FG domains form a molecular brush

SPR measures the binding and release of analytes from surface-tethered ligands. We previously extended this technique to show that noninteracting BSA molecules could be used to determine the average height *h* of a surface layer (22), and validated the BSA-SPR measurements by using atomic force microscopy (AFM) (33). Briefly, the magnitude of the BSA-SPR signal (in terms of resonance units (RU)) gives a measure of *h* because thicker layers give smaller signals than thinner layers. Details of the BSA-SPR method, including calculations of the grafting distance, *g*, for immobilized proteins from the SPR response (using the relation 1300 RU = 1 ng/mm^2^), can be found in previous publications (21,22,33).

**Figure 1:**
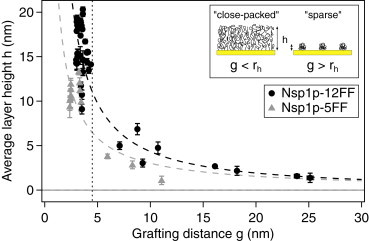
Average layer height, *h*, as a function of grafting distance, *g*, for both Nsp1p FG domain fragments. The vertical dashed line corresponds to their hydrodynamic radii, *r*_*h*_, of 4.5 nm. Flory-Huggins fits predict polyelectrolyte brush behavior. Inset: cartoon description of a molecular brush for *g* < *r*_*h*_ (close-packed) and mushrooms for *g* > *r*_*h*_ (sparse). To see this figure in color, go online.

Two different Nsp1p fragments, Nsp1p-5FF and Nsp1p-12FF, were used in the SPR experiments. Both constructs contain N-terminal 2× Cys-, His_6_-, and S-tags, and have equally spaced FG repeats separated by hydrophilic linker regions. Dynamic light scattering (DLS) gave their hydrodynamic radii (*r*_*h*_) as 4.4 ± 1.0 nm for Nsp1p-5FF and 4.3 ± 1.3 nm for Nsp1p-12FF, although *r*_*h*_ of Nsp1p-5FF may have been slightly overestimated due to polydispersity (Supporting Material). As shown in Fig. 1, surface-tethered Nsp1p layers exhibited a steep increase in layer height, indicating that close packing (*g* < *r*_*h*_) resulted in molecular brush formation (34). The average brush heights were *h*_5*FF*_ = 11.0 ± 1.2 nm, which was smaller than *h*_12*FF*_ = 15.7 ± 2.7 nm. Importantly, the average FG repeat volume densities were 0.058 FG/nm^3^ (Nsp1p-5FF) and 0.062 FG/nm^3^ (Nsp1p-12FF), respectively, reproducing the anticipated FG repeat density within the yeast NPC (0.08 FG/nm^3^) (35).

### Binding of Kap*β*1, NTF2, and W7A-NTF2 to Nsp1p FG brushes

**Figure 2:**
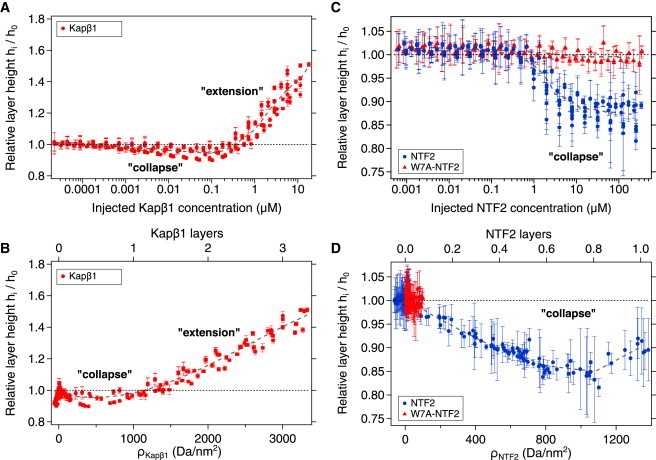
Conformational response of close-packed Nsp1p-12FF layers upon binding Kap*β*1, NTF2, and W7A-NTF2. (*A–D*) The relative layer height is shown as a function of (*A*) injected Kap*β*1 bulk concentration, (*B*) surface density and equivalent number of bound Kap*β*1 layers, (*C*) injected NTF2 or W7A-NTF2 bulk concentration, and (*D*) NTF2 or W7A-NTF2 surface density and equivalent number of bound layers. Collapse was not observed for W7A-NTF2 binding. Error bars are ± SD. Dashed gray lines represent a sliding average. To see this figure in color, go online.

Fig. 2 *A* shows the close-packed Nsp1p-12FF brush height, *h*_*i*_, normalized by its initial height, *h*_0_, measured after each consecutive injection, *i*, of Kap*β*1. Brush collapse was observed below 100 nM Kap*β*1, followed by a 50% layer extension in 10 *μ*M Kap*β*1 that reached a height of ∼24 nm. This height indicated that the Nsp1p brush was fully occupied by approximately three Kap*β*1 layers (Supporting Material) based on the ∼10 nm hydrodynamic diameter of Kap*β*1 and a bound surface density, *ρ*_Kap*β*1_, of 3330 Da/nm^2^ (where one Kap*β*1 layer = 1000 Da/nm^2^) (22) (Fig. 2 *B*). This was comparable to how Kap*β*1 binds the FxFG domains of Nup214, Nup62, and Nup153 (21).

We then compared NTF2’s interaction with the Nsp1p-12FF brush and its interaction with Kap*β*1, using as a negative control the NTF2 W7A mutant (W7A-NTF2), in which FG Nup binding is impaired (28). Fig. 2 *C* shows that the change in layer height was negligible for both proteins at low concentrations. For wild-type NTF2, a decrease in layer height started at an NTF2 concentration of ∼1 *μ*M, reached a ∼12% (2 nm) reduction at physiological concentrations (∼20 *μ*M) (36), and reached an overall reduction of 15% at the highest concentration tested (∼270 *μ*M). No change in layer height was observed with the W7A mutant, even at extremely high concentrations (up to ∼300 *μ*M), consistent with previous studies showing that a reduced avidity of the W7A mutant for Nsp1p impaired NTF2-mediated nuclear import of RanGDP (28,37). Whereas up to 1400 Da/nm^2^ or approximately one layer of wild-type NTF2 was bound (where one layer of NTF2 = 1342 Da/nm^2^), less than 100 Da/nm^2^ of W7A-NTF2 was bound (equivalent to ∼0.05 layers) at the highest injected bulk concentration (Fig. 2 *D*).

### Binding avidity of Kap*β*1, NTF2 and W7A-NTF2 to Nsp1p FG brushes

**Figure 3:**
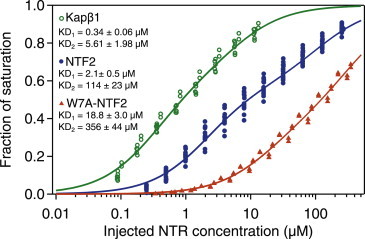
Semi-log plot showing the equilibrium binding of Kap*β*1 (*open circles*), NTF2 (*solid circles*), and W7A-NTF2 (*triangles*) to Nsp1p-12FF brushes. The data were normalized by the maximum binding capacity (fraction of saturation) and are shown as a function of injected bulk NTR concentration. Solid lines represent the average two-component Langmuir isotherm for Kap*β*1, NTF2, and W7A-NTF2, respectively. To see this figure in color, go online.

Fig. 3 shows the equilibrium binding responses of Kap*β*1, NTF2, and W7A-NTF2 to Nsp1p-12FF. Because in each case single isotherm fits proved suboptimal (indicating there was multivalent binding), we analyzed these data by using a two-component Langmuir isotherm. For Kap*β*1, a high-avidity species with KD_1_ = 336 ± 63 nM represented tight binding at high FG repeat density in close-packed Nsp1p FG brushes, whereas moderate binding at KD_2_ = 5.6 ± 2.0 *μ*M was consistent with reduced binding due to preoccupancy of Kap*β*1 and a limited access to FG repeats within the layer (21). NTF2 gave dissociation constants of KD_1_ = 2.1 ± 0.5 *μ*M and KD_2_ = 114 ± 23 *μ*M, which were similar for Nsp1p-5FF and Nsp1p-12FF (Fig. S5). KD_2_ indicated that a nonnegligible fraction of NTF2 bound to the Nsp1p FG domains much more weakly than the known primary physiological interaction (28,36). In comparison, a marked reduction in binding was observed for W7A-NTF2 that had KD_1_ = 18.8 ± 3.0 *μ*M and KD_2_ = 356 ± 44 *μ*M. In spite of KD_1_ being about an order of magnitude weaker than wild-type NTF2, the remaining low avidity given by KD_2_ for W7A-NTF2 indicated the existence of less specific FG binding sites on NTF2, as predicted by NMR (38) and computational studies (39,40).

### Analyses of multivalent binding kinetics to Nsp1p FG brushes

Although an equilibrium binding analysis provides thermodynamic information (e.g., on the stability of the NTR-Nsp1p complex), the temporal transition between bound and unbound NTR forms depends on the kinetic on- and off-rates (*k*_on_ and *k*_off_, respectively). Therefore, we applied the method of Svitel et al. (26) to identify fast- and slow-binding populations of each respective NTR, as was previously done for Kap*β*1 (21). In this manner, we could obtain a more resolved distribution of KDs by knowing *k*_on_ and *k*_off_.

**Figure 4:**
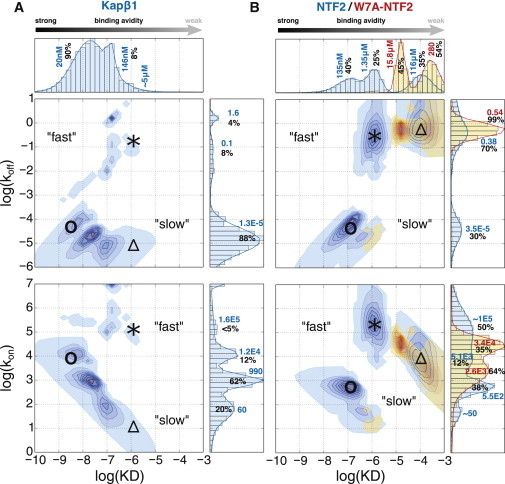
(*A* and *B*) Multivalent kinetic analysis of (*A*) Kap*β*1 and (*B*) NTF2/W7A-NTF2 binding to Nsp1p-12FF brushes. Two-dimensional interaction maps of kinetic on- and off-rates (*k*_on_ and *k*_off_, respectively) are shown with their derived equilibrium binding constant, KD. The fractional abundance of different kinetic states is indicated by the color intensity and the sum over all values in a given axis is shown as accompanying histograms (*top and right panels*). Each distribution is given in percent of the total sum and their main values are in bold. For Kap*β*1, the different kinetic species are labeled with ○ (high-avidity slow phase), ^∗^ (low-avidity fast phase), and Δ (low-avidity slow phase). For NTF2, the different kinetic species are labeled with ○ (high-avidity slow phase), ^∗^ (mid-avidity fast phase), and Δ (low-avidity fast phase). Values corresponding to W7A-NTF2 are depicted in red. Units are s^−1^ and s^−1^M^−1^ for *k*_off_ and *k*_on_, respectively.

Fig. 4 *A* shows that Kap*β*1 binding to Nsp1p-12FF features a broad distribution of affinities ranging from nanomolars to micromolars. Except for the peak at ∼20 nM, the KDs at ∼150 nM and ∼3–5 *μ*M were in good agreement with the KDs from the equilibrium binding analysis (Fig. 3). At low Kap*β*1 concentrations, a high-avidity slow phase (○) commenced at *k*_on_ = 1.2 × 10^4^ s^−1^M^−1^, *k*_off_ = 1.3 × 10^−5^ s^−1^, resulting from a long-lived half-life of *t*_1/2_ ≈ 15 h (where *t*_1/2_ = ln (2)/*k*_off_). Increasing the concentration toward 10 *μ*M Kap*β*1 led to a steady reduction in *k*_on_ to ∼60 s^−1^M^−1^ (Δ), giving rise to lower-avidity interactions (increasing KD) that coincided with the emergence of a low-avidity fast phase (^∗^) having a fast *k*_on_ (∼1.6 × 10^5^ s^−1^M^−1^) and a fast *k*_off_ (0.1–1.6 s^−1^), where now *t*_1/2_ = 430 ms to 7 s. These results were consistent with Kap*β*1 binding to human FG domains observed previously (21), and were indicative of an overall reduction in avidity resulting from 1) a reduction of available FG repeats, 2) poor penetration due to Kap*β*1 occupancy and crowding, 3) a reduced mobility of flexible FG chains due to Kap*β*1 binding, and 4) steric repulsion due to FG chain extension. In this respect, the coexistence of both slow (low *k*_off_) and fast phases (high *k*_off_) at *μ*M Kap*β*1 concentrations indicated that the quantity and/or accessibility of the FG repeats was reduced as Kap*β*1 accumulated in the layer.

Fig. 4 *B* summarizes the distribution of *k*_on_ and *k*_off_ obtained for the binding of NTF2 and W7A-NTF2 to Nsp1p-12FF. For NTF2, the obtained KDs gave distinct peaks at ∼100 nM, ∼1 *μ*M, and ∼100 *μ*M. Overall, we identified three distinctive kinetic species: 1) a high-avidity slow phase (**○**) with low *k*_on_ (∼500 s^−1^M^−1^), low *k*_off_ (∼3.5 × 10^−5^ s^−1^), and long half-life of *t*_1/2_ ≈ 5.5 h; 2) a mid-avidity fast phase (^∗^) with high *k*_on_ (∼10^5^ s^−1^M^−1^), high *k*_off_ (between 0.3–10 s^−1^), and short *t*_1/2_ of ∼70 ms to 2 s; and 3) a low-avidity fast phase (Δ) consisting of a reduced *k*_on_ (∼5100 s^−1^M^−1^) and a similar high *k*_off_ compared with the mid-avidity fast phase. The apparent bimodal distribution of *k*_off_ was consistent with the presence of two major complexes with different stabilities. Although high micromolar-to-millimolar affinities are often considered as nonspecific, they are relevant for NTRs binding to individual FG repeats during transit through the NPC transport channel because of their high off-rates (19). Except for the low KD range peaking around ∼100 nM, the KD distribution obtained from the multivalent kinetic analysis was in good agreement with the KDs from the equilibrium binding analysis (Fig. 3). Overall, the Nsp1p-5FF and Nsp1p-12FF FG domain constructs gave very similar results (Fig. S6).

By comparison, a substantially weaker complex formed during W7A-NTF2 binding to Nsp1p FG repeats, as underscored by the absence of a high-avidity slow phase (Fig. 4 *B*). This indicated binding affinities of approximately 16 *μ*M and 300 *μ*M, in good agreement with the Langmuir isotherm analysis (Fig. 3). Hence, W7A-NTF2 still bound to the FG domains via a number of other putative sites (38,40), although its primary FG repeat binding site at Trp7 is impaired. Conversely, this confirmed that Trp7 is required for the high-avidity, slow-phase binding of wild-type NTF2 that leads to the collapse of close-packed Nsp1p FG domains (Fig. 2 *C*).

### Promiscuous binding of Kap*β*1 and NTF2 to Nsp1p FG brushes

**Figure 5:**
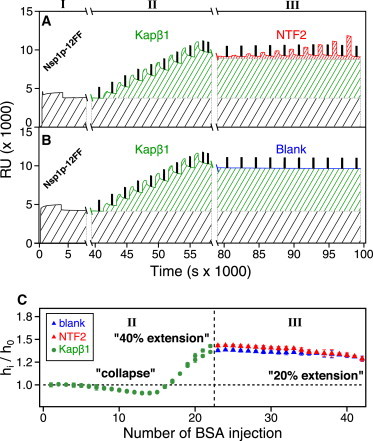
(*A* and *B*) Representative data showing the SPR response of (*A*) NTF2 binding (*red shaded area*) and (*B*) blank PBS (*blue shaded area*) injections to Kap*β*1-preloaded (*green shaded area*) Nsp1p-12FF brushes (*black shaded area*), respectively. For clarity, the black spikes correspond to BSA injections. In both cases, Kap*β*1 binding to Nsp1p-12FF is long-lived with a considerable occupancy. In comparison, NTF2 binding to Nsp1p-12FF is short-lived with a far lower occupancy. (*C*) Corresponding height changes in a Kap*β*1-preloaded Nsp1p-12FF layer after NTF2 injections (*vertical dashed line*). The layer transitions from a 40% extension at 15 *μ*M Kap*β*1 to a 20% extension in 270 *μ*M NTF2. Note the similarity in layer height when blanks (i.e., PBS) are injected.

We then investigated how binding promiscuity would affect Kap*β*1 and NTF2 binding. Generally, resolving how two different analytes interact simultaneously with surface-tethered ligands is not straightforward in SPR. However, in these circumstances, it was permissible to analyze this because the majority of Kap*β*1 molecules that bind and occupy Nsp1p were far longer lived than NTF2 (Fig. 4). These effects are readily visible in the representative data shown in Fig. 5. For clarity, one measurement contained the binding of up to ∼15 *μ*M Kap*β*1 followed by increasing titrations of NTF2 (Fig. 5 *A*). Another measurement contained the binding of up to ∼15 *μ*M Kap*β*1 followed by blank injections (i.e., PBS buffer; Fig. 5 *B*).

After eluting for 2230 s past the final Kap*β*1 injection, ∼2.5 layers or 80% of Kap*β*1 remained bound in the Nsp1p brush that had extended by 40% over its initial height (Fig. 5 *C*). Surprisingly, both NTF2 (Fig. 5 *A*) and blank (Fig. 5 *B*) injections elicited the same height change from this Kap*β*1-preloaded brush, which reduced to a 20% extension at the highest NTF2 concentration (i.e., 270 *μ*M; Fig. S7). This indicated that NTF2 binding did not significantly impact the structural integrity of Nsp1p in the presence of strongly bound Kap*β*1, which clearly had very slow off-rates. Indeed, if NTF2 binding facilitated Kap*β*1 dissociation (washing out of bound Kap*β*1), one would anticipate a more marked reduction in layer height (Fig. 2 *C*). We then subtracted the intrinsic slow phase of Kap*β*1 (Fig. 5 *B*) from the combined Kap*β*1/NTF2 SPR signal (Fig. 5 *A*) to decouple and isolate the signal of promiscuously bound NTF2 (Fig. S8).

**Figure 6:**
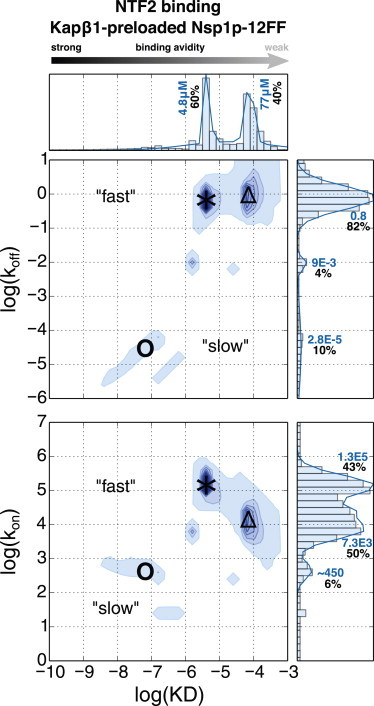
Multivalent kinetic analysis of NTF2 binding close-packed Nsp1p FG domains preloaded with Kap*β*1. Two-dimensional interaction maps of kinetic on- and off-rates (*k*_on_ and *k*_off_, respectively) are shown in relation to the equilibrium binding constant KD. The fractional abundance of different kinetic states is indicated by the color intensity and the sum over all values in a given axis is shown as accompanying histograms (*top and right panels*). Different kinetic species are labeled with ○ (high-affinity slow phase), ^∗^ (mid-affinity fast phase), and Δ (low-affinity fast phase). Each distribution is given in percent of the total sum and their main values are depicted in bold. Units are s^−1^ and s^−1^M^−1^ for *k*_off_ and *k*_on_, respectively. To see this figure in color, go online.

Subsequent multivalent analyses revealed that the difference between promiscuous NTF2 binding in the presence of Kap*β*1 compared with NTF2 binding pristine Nsp1p brushes was significant. As shown in Fig. 6, NTF2 binding avidity was dominated by weak KDs at 4.8 *μ*M and 77 *μ*M, where 80% of the bound fraction exhibited fast *k*_off_ (i.e., 1 s^−1^; *t*_1/2_ = 70 ms; see Fig. S9 for equilibrium binding analyses). This was consistent with a lack of significant competition between the already bound Kap*β*1 and the added NTF2. Hence, an overall trend toward faster and more transient interactions of NTF2 was observed when Kap*β*1 was present in the Nsp1p brush. This corresponded to 0.06 layers of NTF2 at the highest injected concentration of 270 *μ*M.

## Discussion

### Nsp1p FG domains form a molecular brush

FG domain morphology and its response to binding are strongly dependent on surface tethering (41) because this imposes a surface boundary that limits NTR occupancy (21). Due to lateral crowding, entropic effects dominate over, but do not preclude, competing enthalpic interactions between chains (i.e., cohesion), resulting in Nsp1p forming a molecular brush. Importantly, the close agreement between the FG repeat density (∼0.06 FG/nm^3^) obtained in this study and that obtained in yeast NPCs (0.08 FG/nm^3^) (35) makes it an attractive in vitro system in which to study the functional properties of FG Nups when they are binding different NTRs.

### NTF2 binding leads to Nsp1p brush collapse and Kap*β*1 drives its expansion

Our results demonstrated that NTF2 and Kap*β*1 binding to FG regions of Nsp1p influenced the brushes very differently. Surprisingly, the Nsp1p brush exhibited collapse at even the highest NTF2 concentrations used. Within the physiological range (∼20 *μ*M NTF2), the collapse was ∼12% of the initial layer height, with the bound content corresponding to effectively one monolayer of NTF2. In comparison, Kap*β*1 binding was characterized by a nonmonotonic response that collapsed the Nsp1p brush at low nanomolar concentrations (7), followed by a self-healing extension (22) at physiological (*μ*M) concentrations. This was due to an increasing occupancy of Kap*β*1, which formed multilayers within the brush, and was consistent with SPR measurements of Kap*β*1 binding to Nup214, Nup62, and Nup153 (21).

These data show a size-dependent effect that differentiated NTF2 (small) from Kap*β*1 (large), and support the theory of Opferman et al. (42,43), which predicts that binding-induced conformational changes in polymer brushes depend on the nanoparticle size and the interaction energy with the polymer. Thus, changes in brush height originate from competition between the binding energy of nanoparticles to the polymer, favoring collapse, and the confinement entropy of the polymers, promoting extension. Although Kap*β*1 showed a higher avidity for FG repeats than NTF2, its binding at physiological concentrations favored layer extension because of its relatively large volume, which impacts the entropy of the FG domains. Because NTF2 is smaller, its binding favors collapse over extension, although the latter may be possible at higher (but nonphysiological) concentrations. By contrast, W7A-NTF2 did not collapse the brush because it only bound very weakly to Nsp1p.

Our results are consistent with measurements of Kap95p (yeast importin*β*) binding to Nsp1p residues 2–601 in layers with comparable surface grafting densities (∼4 nm) (44). The two-component KD we obtained by SPR (340 nM and 5.6 *μ*M) was indistinguishable from the KD values (320 nM and 5.3 *μ*M) obtained by ellipsometry (44). Notwithstanding methodological differences, the SPR-measured height increase was also comparable to the ∼4 nm Nsp1p layer extension seen with 5 *μ*M Kap95p using a quartz crystal microbalance with dissipation (44). Coincidentally, the average FG repeat concentration of 106 ± 18 mM (i.e., 0.064 FG repeats/nm^2^) reported by Eisele et al. (44) was equivalent to the FG repeat density obtained here. Indeed, the transition from brush collapse into extension we found at 0.2 *μ*M Kap*β*1 (Fig. 2 *A*) may explain why AFM did not detect Nsp1p collapse at similar concentrations of Kap95p. On a more technical note, our SPR method is limited to static height measurements and cannot capture dynamic reversible collapse events of single FG Nups, such as those obtained by single-molecule fluorescence (45).

### Kinetic analysis of multivalent binding

Understanding how NTF2 and Kap*β*1 bind Nsp1p separately provides benchmarks for the avidity that is manifest from multivalent interactions with proximal FG domains. Overall, both NTF2 and Kap*β*1 formed more than one complex with the Nsp1p FG domains. This was evident from the existence of multiple KDs, as obtained from equilibrium binding analyses and the distribution of *k*_on_ and *k*_off_ obtained from multivalent kinetic analyses. The structural basis of this behavior is likely complicated, but can be rationalized given that a single Nsp1p chain can bind multiple copies of the same NTR (one to many) or several FG domains can bind simultaneously to a single NTR (many to one), or a combination of both characteristics could occur. This is consistent with the behavior of intrinsically disordered proteins (25).

The kinetics of Kap*β*1 binding to Nsp1p was similar to that observed for its binding to human Nup214, Nup62, Nup98, and Nup153 (21). This was characterized by ∼90% of bound Kap*β*1 exhibiting stronger and longer complex lifetimes (low *k*_off_) accompanied by a minority exhibiting high off-rates associated with binding at the Nsp1p periphery (Fig. 4). In contrast, NTF2 binding was more transient, with 70% of bound molecules showing fast off-rates and 99% of W7A-NTF2 being in this fast regime. Except for the high-avidity complex formed at KD = 135 nM, the ∼1 to 2 *μ*M and ∼100 *μ*M KDs obtained for NTF2 from both equilibrium and kinetic analyses were consistent with previous single-value estimates (28,36). Because NTF2 has fewer FG binding sites and is smaller in size than Kap*β*1, its multivalent binding kinetics may be dominated less by in-layer crowding and more by local structural effects, especially since NTF2 occupancy only reached one layer in the Nsp1p brush even at the highest titrates (Fig. 2 *D*). Its low- and high-avidity modes may result from the occupation of one or two FG binding sites on the NTF2 dimer, respectively. Alternatively, NTF2 could bind two FG repeats on a single Nsp1p chain or to single FG repeats on two different Nsp1p chains. We speculate that the latter interaction would be more favored energetically, since the former would more considerably restrict the Nsp1p conformation. Irrespective of the precise mechanism involved, impairing the primary FG interaction sites on the W7A mutant impacted both interactions.

### Promiscuous binding of NTF2 to Nsp1p in the presence of Kap*β*1

Preloading Nsp1p brushes with Kap*β*1 had a dramatic influence on the binding of NTF2. Binding Kap*β*1 to Nsp1p should reduce its flexibility (so Nsp1p becomes increasingly rigid (7,44)) and also reduce the availability of free FG repeats. Consequently, the avidity of NTF2 would be weakened by the extent to which Kap*β*1 is bound. Because Kap*β*1 binds more strongly to Nsp1p than NTF2, its occupancy is higher, forcing the layer to extend and making it harder for NTF2 molecules to penetrate the Kap*β*1-dominated volume. Under these conditions, kinetic analysis indicated that the two stronger, specific interaction modes identified with NTF2 alone were altered in a manner similar to that observed when the W7A mutant bound to a pristine Nsp1p brush. Here, the strongest mode was essentially eliminated, whereas the avidity of the weaker mode was reduced and the weakest (probably nonspecific) binding was not altered greatly. Reduced Nsp1p chain flexibility may increase the entropic cost of binding two Nsp1p chains to a single NTF2 dimer and thus inhibit formation of the strongest binding mode. Similarly, the entropic penalty associated with binding a single chain would also increase, resulting in decreased avidity and hence an increase in the bound NTF2 population with high off-rates (i.e., 80%; Fig. 6).

### Kap*β*1 contributes to the NPC barrier function and promotes fast NTF2 kinetics

Recently, it was proposed that Kap*β*1 is an integral, bona fide constituent of the NPC barrier, which is often assigned to the FG Nups alone, and that Kap*β*1 contributes to modulating both mechanistic and kinetic aspects of NPC barrier functionality (21). Here, the stronger and longer-lived FG domain-binding interactions exhibited by Kap*β*1 compared with those of NTF2 provide support for such a Kap-centric barrier mechanism (21,22). In this context, promiscuous binding of Kap*β*1 may be essential to maintain NPC barrier function by increasing the rigidity of the FG domain layer (7,44) to increase the barrier against molecules that bind nonspecifically (23,46). Indeed, studies show that the immobile fraction of Kap*β*1 (∼100 molecules/pore) is substantially larger than that of NTF2 (∼6 molecules/pore) (20).

**Figure 7:**
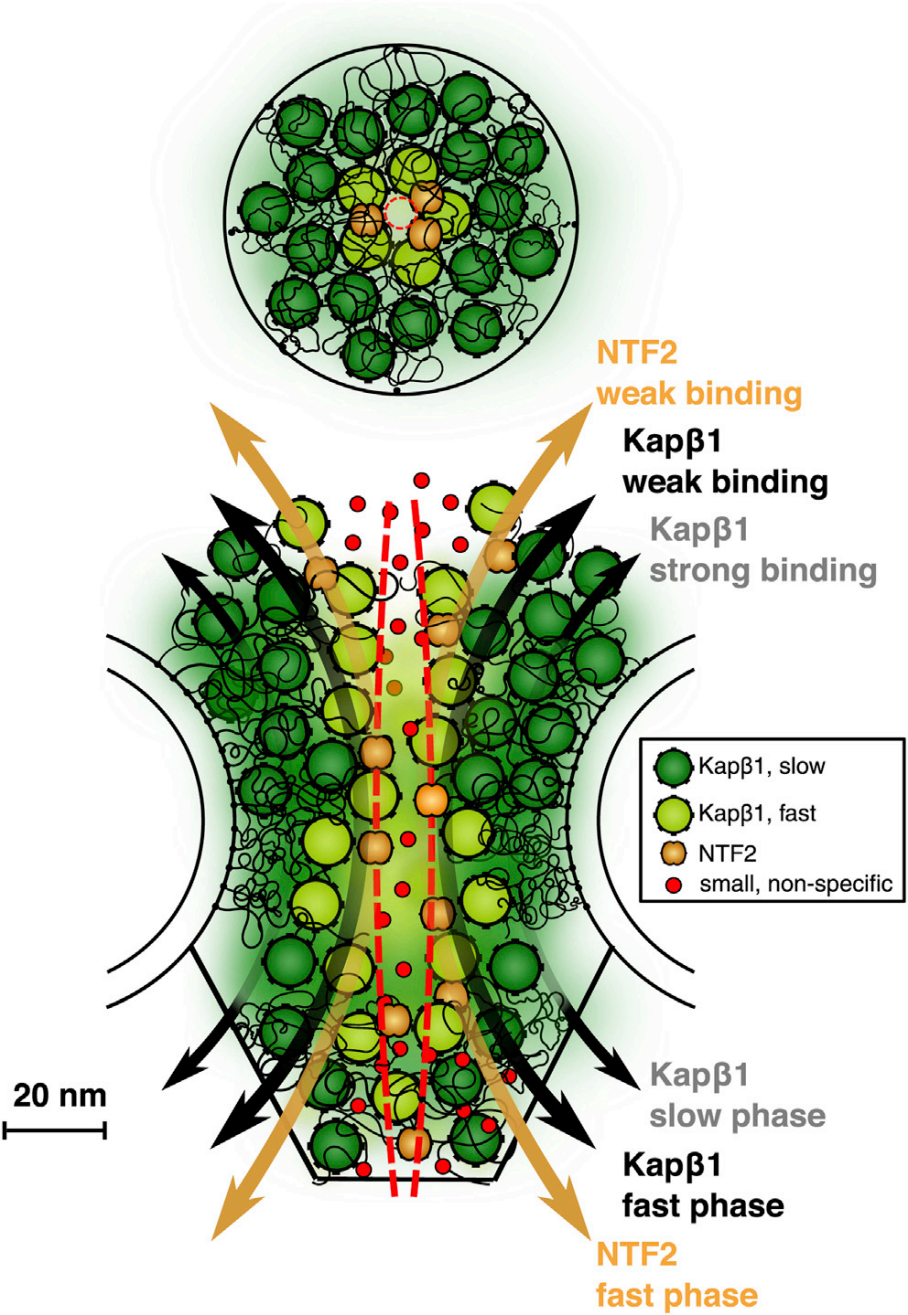
Kap-centric barrier model showing how different NTRs may share contiguous spatial and temporal routes through the NPC. Strongly bound Kap*β*1 molecules (slow) occupy the FG Nups and form integral constituents of the barrier mechanism. This crowding promotes the facilitated diffusion of NTF2 and a smaller fraction of Kap*β*1 (fast) through a central conduit bearing a reduced density of FG repeats. To see this figure in color, go online.

As illustrated in Fig. 7, the presence of slow-phase Kap*β*1 would hinder and limit how far NTF2 penetrates into the FG layer, thereby counterbalancing NTF2-mediated FG domain collapse. Accordingly, the fast interaction kinetics (high *k*_off_) of NTF2 could promote selective diffusion along the peripheral regions of the engorged FG domains in a manner that is contiguous with the fast Kap*β*1 phase (21), such as by a reduction of dimensionality (23,47). Indeed, both NTRs appear to traverse NPCs simultaneously and with similar dwell times of ∼5 ms (48,49). Consistent with the Kap-centric model, in vitro nuclear protein import assays show increased transport rates with increasing Kap*β*1 concentrations (24). We further speculate that decreasing the effective Kap*β*1 concentration or occupancy at the NPC would generate a less effective barrier (i.e., more open, less selective) due to NTF2-mediated FG domain collapse.

A formidable challenge lies in decoupling the diverse pathways that converge on NPCs, constituting the main nucleocytoplasmic transport hub (50). Clearly, the pore channel is crowded (20), and it is essential to know the effective local concentrations (51) of each transport receptor in and around the NPC. It is also crucial to establish how the loading of Kap*α* and specific cargoes influences Kap*β* binding, and the extent to which different NTRs bind preferentially to different FG Nups. In terms of binding promiscuity, this could demarcate not only spatial pathways (52) but also temporal ones. Irrespective of the precise mechanisms involved, promiscuous binding and the influence of Kap*β*1 binding on the off-rate of other NTRs clearly make contributions that one should take into account when formulating precise models of nucleocytoplasmic transport.

## Conclusions

To our knowledge, these results demonstrate for the first time that promiscuous binding of NTRs to FG Nups should influence nucleocytoplasmic transport. This depends on the concentration, size, and binding strength of each NTR. Indeed, some form of hierarchy may exist between different NTRs such that their relative concentrations may impact NPC barrier function. This interpretation departs from the conventional view that the FG Nups alone form the NPC permeability barrier. Rather, we propose that concentrating NTRs in the NPC transport channel also contributes to generating the crowding-based selective barrier function of the pore.

## Supporting Material

Document S1. Supporting Materials and Methods and nine figures

Document S2. Article plus Supporting Material
