# Supplementary material for: Promiscuous Binding of Karyopherinβ1 Modulates FG Nucleoporin Barrier Function and Expedites NTF2 Transport Kinetics
Source: Biophys J. 2015 Feb 17;108(4):918–27. doi: 10.1016/j.bpj.2014.12.041 (PMC4336380; doi:10.1016/j.bpj.2014.12.041)
Supplement: Document S1. Supporting Materials and Methods and nine figures [file mmc1.pdf]

Promiscuous Binding of Karyopherinβ1 Modulates FG Nucleoporin Barrier Function and Expedites NTF2 Transport Kinetics

Raphael S. Wagner[1], Larisa E. Kapinos[1], Neil J. Marshall[2], Murray Stewart[2] and Roderick Y. H. Lim[1*]

[1]Biozentrum and the Swiss Nanoscience Institute, University of Basel, Basel, Switzerland.
[2]MRC Laboratory of Molecular Biology, Cambridge, United Kingdom.

*Correspondence: roderick.lim@unibas.ch

Supporting Material

1. Sequence of FG domain fragments and purity of expressed proteins

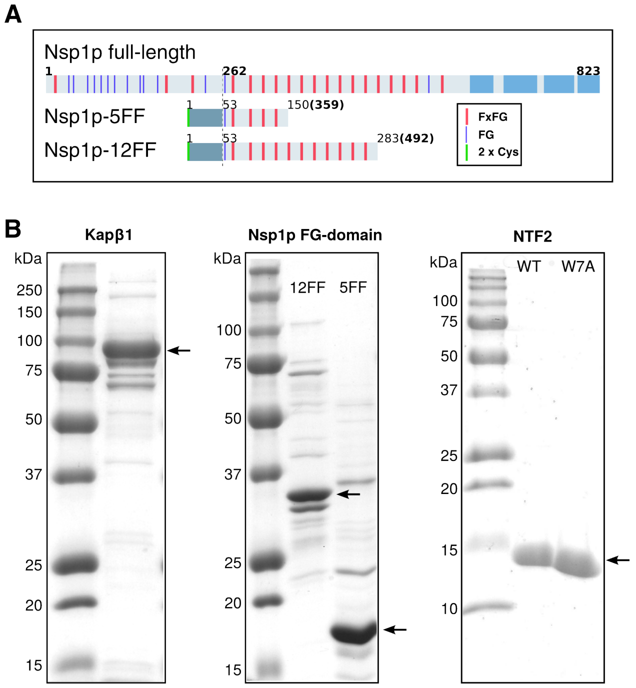

Figure S1. (A) Both FG domain fragments used in this study, Nsp1p-5FF and Nsp1p-12FF, contain regularly spaced FSFG repeats. Residue numbers in bold correspond to wild-type Nsp1p. The fragments contain a 52-residue 2xCys-/6xHis-/S-tag at their N-termini. The structured C-terminal domains of full-length Nsp1p are depicted in light blue. **(B)** 6% PAGE (0.1% SDS) of Kapβ1, 15% PAGE (0.1% SDS) of Nsp1p FG-domain fragments Nsp1p-12FF and Nsp1p-5FF, and 15% PAGE (0.1% SDS) of NTF2 and W7A-NTF2, respectively.

2. Regeneration of a Kapβ1 loaded Nsp1p-12FF layer

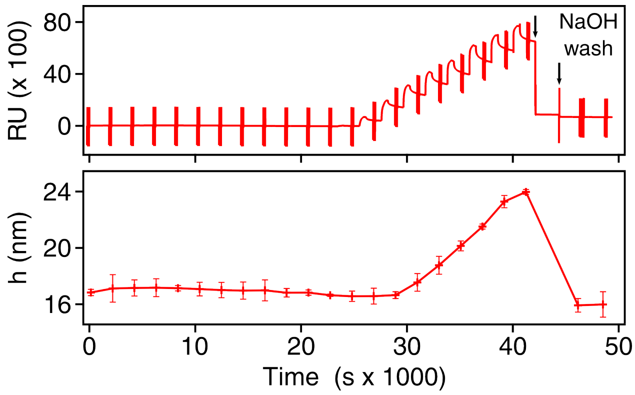

Figure S2. Nsp1p-12FF-bound Kapβ1 was removed after two short injections (30 s) of 0.2 M NaOH in PBS (pH ~13). This verifies that Kapβ1 is not covalently bound to the gold SPR sensor. Top: SPR response reaching baseline indicating Kapβ1 removal. Bottom: Corresponding Nsp1p-12FF layer height that is restored to its initial value after Kapβ1 removal.

3. Measuring FG domain layer height

1 % (w/v) non-interacting BSA molecules were injected and the corresponding SPR response (RU) was used to estimate a layer height, h, as described in detail by Schoch, *et al.* (1). The experimental parameters are summarized in Fig. S3.

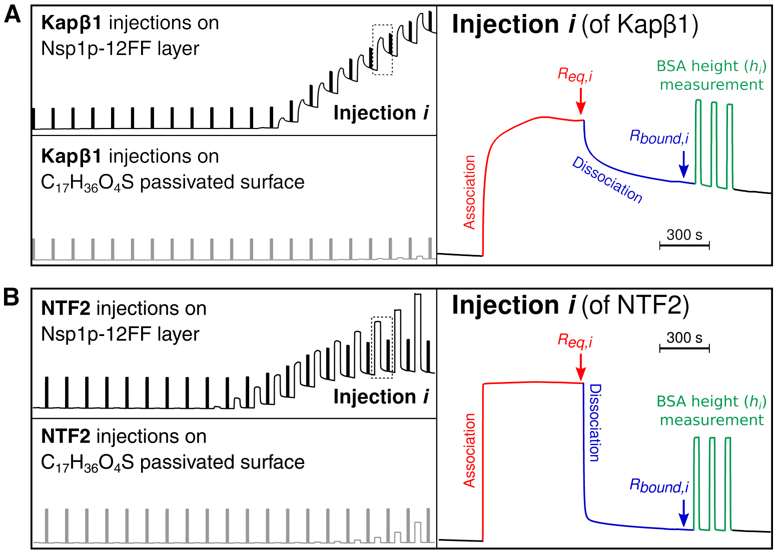

Figure S3. (A) Kapβ1 and **(B)** NTF2 were injected into Nsp1p functionalized and $C_{17}H_{36}O_4S$ passivated channels (left top and bottom, respectively). NTR-Nsp1p-12FF interactions consisted of an association (red) and dissociation (blue) phase followed by BSA injections (green). $R_{eq,i}$ corresponds to the SPR equilibrium binding response from each NTR injection of bulk concentration c_i with respect to the baseline (i.e., response at t = 0 s). $R_{bound,i}$ represents the SPR response for bound NTR after 480 s of dissociation (c_i = 0). Three consecutive BSA injections were used to measure the average layer height at layer occupancy $R_{bound,i}$. The BSA signal (green) returns to baseline indicating a lack of binding to Nsp1p FG domains, which is a prerequisite for reliable height measurements.

4. Multivalent binding kinetic analysis

The concept of surface heterogeneity as introduced by Svitel, *et al*. (2) was used to estimate the binding kinetics of Kapβ1 interacting with Nsp1p FG-domains as explained in detail by Kapinos *et al*. (3). The same approach was applied to NTF2 (and W7A-NTF2) binding using a simplified kinetic model assuming pseudo first-order kinetics:

$$L + D_S \underset{k_{off,\,i}}{\overset{k_{on,\,i}}{\rightleftharpoons}} LD_S \qquad \textbf{(S1)}$$

Here, L is the number of free binding sites, D_s is the NTF2 dimer concentration in solution and LD_s are binding sites occupied with dimeric NTF2 molecules.

Multivalent kinetic fits to representative SPR sensograms for NTF2, W7A-NTF2, Kapβ1 and NTF2 on Kapβ1 preloaded Nsp1p-12FF is given in Fig S4. The corresponding kinetic maps are shown in Fig. 4 and Fig. 6 in the main text.

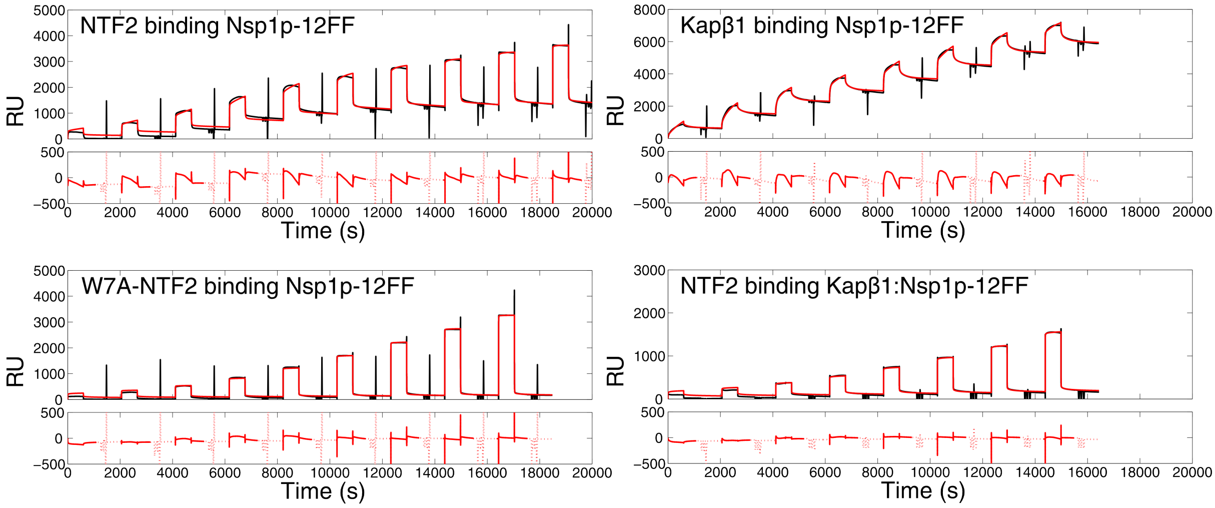

Figure S4. Representative SPR measurements (black), multivalent kinetic fits (red) and residuals shown for NTF2, W7A-NTF2, Kapβ1 and NTF2 binding Kapβ1 preloaded Nsp1p-12FF.

5. Dynamic Light Scattering

Hydrodynamic diameters where obtained by dynamic light scattering (DLS) in PBS with the addition of 1 mM DTT, using a Zetasizer Nano (Malvern). The measured hydrodynamic diameters were d_h = 8.7 ± 2.5 (polydispersity index = 0.3) for Nsp1p-5FF, d_h = 8.6 ± 2.7 (polydispersity index = 0.1) for Nsp1p-12FF, using n_p = 1.45 and n_d = 1.330 as the refractive index for proteins and dispersant, i.e., water; T = 298 K, viscosity = 0.8872 cP (1P = 0.1 Pa·s).

6. Estimation of Kapβ1 and NTF2 layers

The next-neighbor distance, g_{NTR}, between Kapβ1 or (W7A-) NTF2 molecules within the FG-domain layer was estimated as described (4). Accordingly, the number of layers is calculated using

$$Number\ of\ layers = \frac{d_h^2}{g_{NTR}^2} \qquad \textbf{(S2)}$$

where d_h is the respective NTR hydrodynamic diameter. SPR response units (RUs) are converted using the relation 1300 RU = 1 ng/mm^2 (1).

7. Equilibrium analysis of NTF2 binding to Nsp1p-5FF

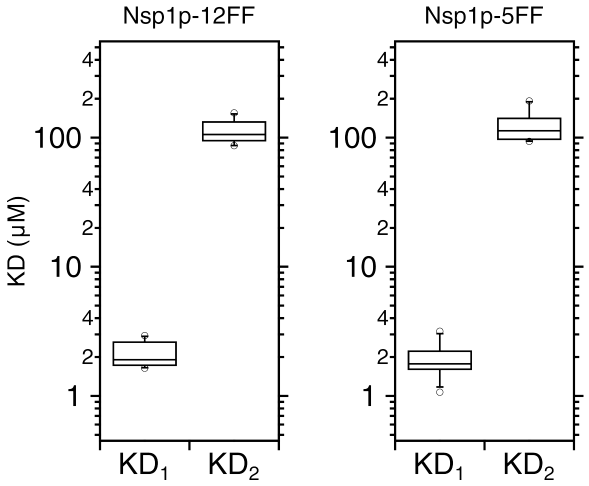

Figure S5. Equilibrium analysis shows that NTF2 binding is equivalent for both Nsp1p-5FF (N = 11) and Nsp1p-12FF (N = 15). The first and third quartiles are represented by box-plots. The median is given by the band inside the box. Whiskers represent the 9th and the 91th percentile. Outliers are plotted as open circles.

8. Multivalent kinetic analysis of NTF2 binding Nsp1p-5FF

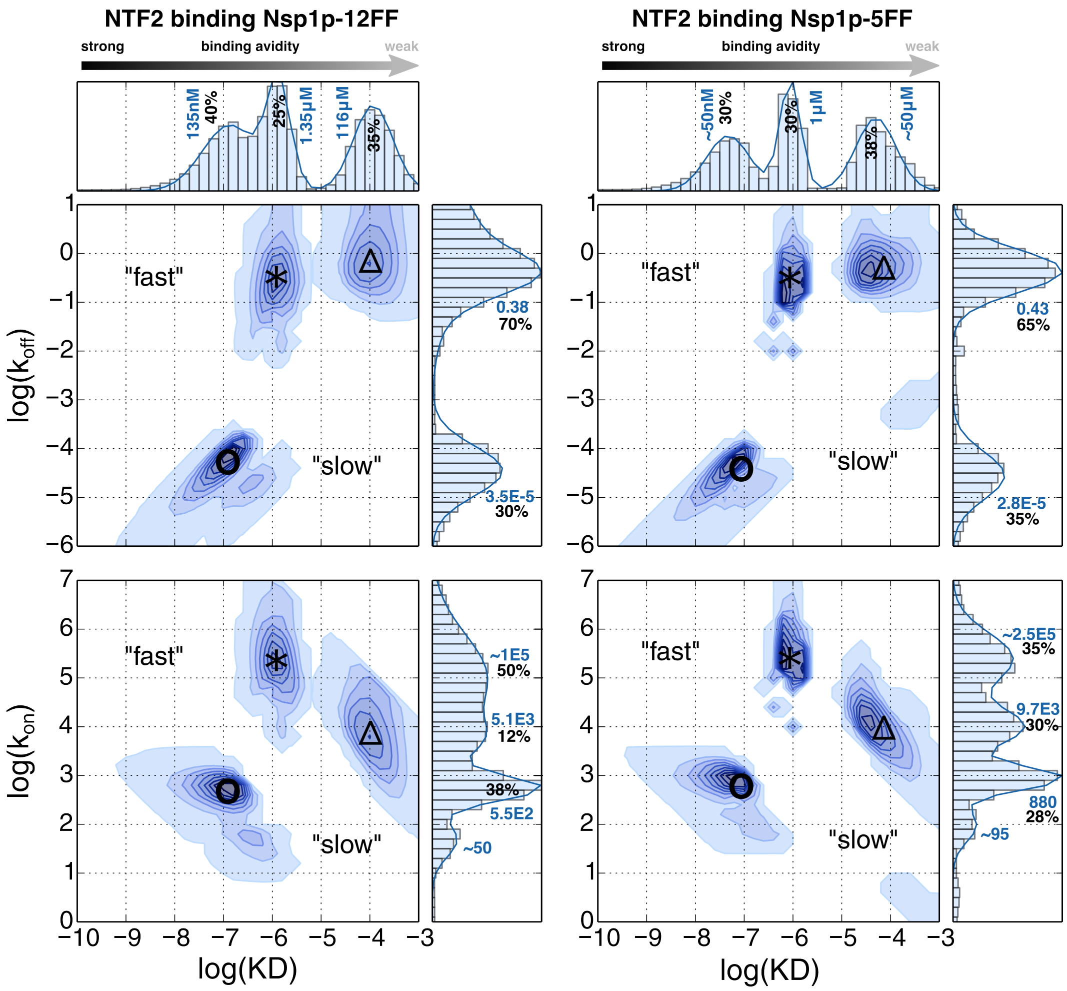

Figure S6. Multivalent binding analysis of NTF2 binding to close-packed layers of Nsp1p-12FF and Nsp1p-5FF. Two-dimensional interaction maps of kinetic on- and off-rates (k_{on}, k_{off}) are shown in relation to the equilibrium binding constant KD. The fractional abundance of different kinetic states is indicated by the color intensity and the sum over all values in a given axis is shown as accompanying histograms (top and right panels). Different kinetic species are labeled with o ("high-avidity slow-phase"), * ("mid-avidity fast-phase") and Δ ("low-avidity fast-phase"). Each distribution is given in percent of the total sum and their main values are depicted in bold. Units for k_{off} and k_{on} are s^{-1} and $s^{-1}M^{-1}$, respectively.

9. NTF2 binding to a Kapβ1-preloaded FG-domain layer

The total layer height did not change when comparing between the intrinsic dissociation of Kapβ1 in the presence and absence of NTF2 binding (Fig. S7).

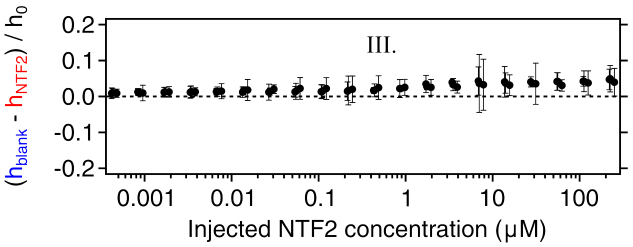

Figure S7. Similar height changes resulted from both NTF2 and blank injections on Kapβ1 preloaded Nsp1p-12FF brushes (normalized by the initial layer height h_0) as a function of NTF2 concentration.

To decouple the NTF2 from the Kapβ1 signal, the slow dissociation phase of Kapβ1 (see Fig. 5B and Fig. S8A) was fitted using a sum of exponential functions describing the dissociation of n = 3 different kinetic species forming binary complexes on the surface, modeled by

$$s(t) = \sum_n A_n e^{-k_{d,n} t} \quad \textbf{(S3)}$$

where $s(t)$ is the experimental SPR signal of Kapβ1, t is time, A_n the individual binding response at t = 0 of each species and $k_{d,n}$ is the apparent kinetic dissociation rate of species n. BSA injections were not included in the fit by using a mask in the fitting procedure.

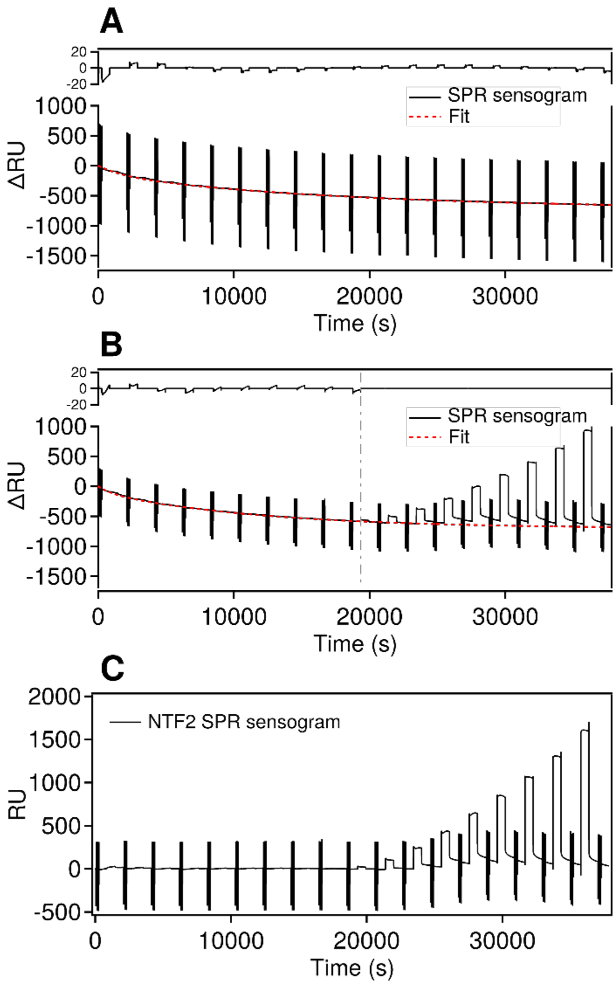

Figure S8. (A) Dissociation of Kapβ1 and fit with residual (top) in the absence of NTF2. **(B)** The dissociation of Kapβ1 was fitted for the first few injections (up to the dashed vertical line) where no significant change in SPR response was measured due to the low concentration of injected NTF2. Residual is shown on top. **(C)** NTF2 binding was isolated by subtracting the fit in (B) from the SPR sensogram of (B).

In a next step, the fit was applied to the dissociation phase of Kapβ1 in the presence of NTF2 binding (Fig. S8B). Finally, the fit obtained for the dissociation of Kapβ1 was subtracted from the total binding signal leaving only the SPR sensogram for NTF2 binding (Fig. S8C). This sensogram was then further analysed using the same protocol as described in Supporting Material 4.

10. Equilibrium analysis of NTF2 binding Kapβ1 preloaded Nsp1p-12FF

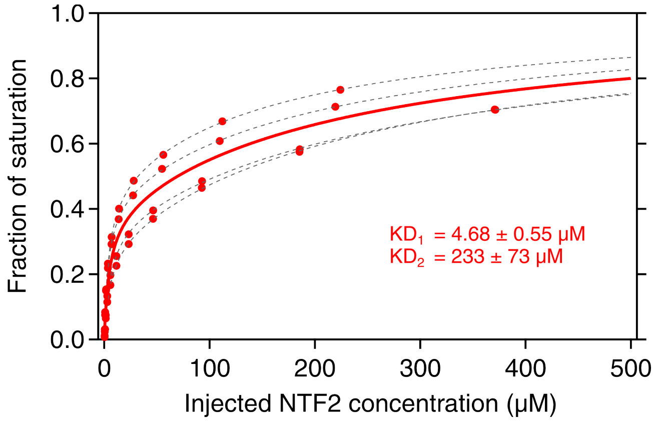

Figure S9. Equilibrium binding analysis of NTF2 binding Kapβ1 preloaded Nsp1p-12FF layers. The SPR equilibrium response was normalized by the maximum binding capacity (fraction of saturation) and shown as a function of injected bulk concentration. Grey dashed lines are individual 2-component Langmuir isotherm fits. The isotherm recalculated from the average KD_1 and KD_2 is shown as a solid line.

Supporting References

1. Schoch, R. L., and R. Y. H. Lim. 2013. Non-interacting molecules as innate structural probes in surface plasmon resonance. *Langmuir*. 29:4068-4076.
2. Svitel, J., H. Boukari, D. Van Ryk, R. C. Willson, and P. Schuck. 2007. Probing the functional heterogeneity of surface binding sites by analysis of experimental binding traces and the effect of mass transport limitation. *Biophys. J.* 92:1742-1758.
3. Kapinos, L. E., R. L. Schoch, R. S. Wagner, K. D. Schleicher, and R. Y. H. Lim. 2014. Karyopherin-centric control of nuclear pores based on molecular occupancy and kinetic analysis of multivalent binding with FG nucleoporins. *Biophys. J*. 106:1751-1762.
4. Schoch, R. L., L. E. Kapinos, and R. Y. H. Lim. 2012. Nuclear transport receptor binding avidity triggers a self-healing collapse transition in FG-nucleoporin molecular brushes. *Proc. Natl. Acad. Sci. USA*. 109:16911-16916.
