# Supplementary material for: Promiscuous Binding of Karyopherinβ1 Modulates FG Nucleoporin Barrier Function and Expedites NTF2 Transport Kinetics
Source: Biophys J. 2015 Feb 17;108(4):918–27. doi: 10.1016/j.bpj.2014.12.041 (PMC4336380; doi:10.1016/j.bpj.2014.12.041)
Supplement: Document S2. Article plus Supporting Material [file mmc2.pdf]

Article

Promiscuous Binding of Karyopherinβ1 Modulates FG Nucleoporin Barrier Function and Expedites NTF2 Transport Kinetics

Raphael S. Wagner,[1] Larisa E. Kapinos,[1] Neil J. Marshall,[2] Murray Stewart,[2] and Roderick Y. H. Lim[1,*]
[1]Biozentrum and the Swiss Nanoscience Institute, University of Basel, Basel, Switzerland; and [2]MRC Laboratory of Molecular Biology, Cambridge, UK

ABSTRACT The transport channel of nuclear pore complexes (NPCs) contains a high density of intrinsically disordered proteins that are rich in phenylalanine-glycine (FG)-repeat motifs (FG Nups). The FG Nups interact promiscuously with various nuclear transport receptors (NTRs), such as karyopherins (Kaps), that mediate the trafficking of nucleocytoplasmic cargoes while also generating a selectively permeable barrier against other macromolecules. Although the binding of NTRs to FG Nups increases molecular crowding in the NPC transport channel, it is unclear how this impacts FG Nup barrier function or the movement of other molecules, such as the Ran importer NTF2. Here, we use surface plasmon resonance to evaluate FG Nup conformation, binding equilibria, and interaction kinetics associated with the multivalent binding of NTF2 and karyopherinβ1 (Kapβ1) to Nsp1p molecular brushes. NTF2 and Kapβ1 show different long- and short-lived binding characteristics that emerge from varying degrees of molecular retention and FG repeat binding avidity within the Nsp1p brush. Physiological concentrations of NTF2 produce a collapse of Nsp1p brushes, whereas Kapβ1 binding generates brush extension. However, the presence of prebound Kapβ1 inhibits Nsp1p brush collapse during NTF2 binding, which is dominated by weak, short-lived interactions that derive from steric hindrance and diminished avidity with Nsp1p. This suggests that binding promiscuity confers kinetic advantages to NTF2 by expediting its facilitated diffusion and reinforces the proposal that Kapβ1 contributes to the integral barrier function of the NPC.

INTRODUCTION

Nuclear pore complexes (NPCs) (1) are intracellular transport hubs that mediate the rapid bidirectional traffic of hundreds of proteins, ribonucleoproteins, and metabolites across the nuclear envelope (2). Each NPC contains a 50-nm-diameter central channel (3) through which only molecules smaller than ~40 kDa (4) or ~5 nm in size (5) can diffuse passively (6). The movement of larger molecules is impaired by a permeability barrier generated by ~200 intrinsically disordered phenylalanine-glycine (FG)-rich nucleoporins (FG Nups) that are tethered to the NPC transport channel surface. Although the precise mechanism by which the barrier is generated in vivo has not been resolved, in vitro the FG Nups collectively resemble molecular brushes (7,8), supramolecular hydrogel meshworks (9–11), or both (12).

The translocation of selective cargoes through NPCs is mediated by a range of soluble nuclear transport receptors (NTRs) (13). These include members of the karyopherin family (Kaps) (14), such as the 97 kDa import receptor karyopherinβ1 (Kapβ1 or importinβ) (15), which recognizes specific cargoes either directly or via an adaptor Kapα. Kapβ1 contains several FG repeat binding pockets that exert multivalent binding interactions with the FG Nups (15–17). Multivalency (18) leads to an enhanced binding affinity through avidity (19). In vivo, each NPC contains as many as 100 Kapβ1 molecules at steady state (20) as a result of Kapβ1 binding to multiple FG Nups, and this would increase molecular crowding substantially. Moreover, Kapβ1 binding has been demonstrated to alter the conformation of four different human FG Nups (Nup214, Nup62, Nup98, and Nup153) in vitro (21,22). Such conformational behavior is nonmonotonic (i.e., nonlinear) and depends on Kapβ1 concentration, such that FG Nup brushes collapse at low nM Kapβ1 concentrations (7) and re-extend at higher μM physiological Kapβ1 concentrations (21,22). As a result, Kapβ1 occupancy within the FG Nups attenuates the binding avidity of incoming Kapβ1 molecules and expedites their dissociation kinetics by reducing the number of available FG repeats (21,22). This is evident in NPC-inspired biomimetic systems (23) and provides a plausible explanation for the dependence of transport efficiency on Kap concentration in permeabilized cell assays (24).

How the binding of Kapβ1 to FG Nups impacts NPC barrier function and influences the binding of other NTRs to FG Nups remains poorly understood. Indeed, such binding promiscuity extends beyond the FG Nups and more generally is relevant to how intrinsically disordered proteins can bind multiple partners simultaneously (25). Here, we apply surface plasmon resonance (SPR) to investigate the effect of

Submitted August 18, 2014, and accepted for publication December 19, 2014.

*Correspondence: roderick.lim@unibas.ch

This is an open access article under the CC BY license (http://creativecommons.org/licenses/by/4.0/).

Editor: Daniel Muller.

© 2015 The Authors

0006-3495/15/02/0918/10 $2.00

http://dx.doi.org/10.1016/j.bpj.2014.12.041

binding promiscuity by measuring the multivalent interaction kinetics (26), equilibrium avidities, and in situ associated conformational changes that occur in Nsp1p when nuclear transport factor 2 (NTF2) and Kapβ1 are bound, both separately and together. NTF2 is an essential homodimeric 30 kDa transport receptor that imports the GTPase Ran from the cytoplasm into the nucleus (27). Although both NTRs exhibit avidities that vary depending on their occupancy within Nsp1p, our data show a size-dependent effect that differentiates NTF2 (small) from Kapβ1 (large). Whereas increasing Kapβ1 from low to physiological concentrations drove the Nsp1p brush from collapse to re-extension, NTF2 caused only collapse. As a control, brush collapse was not seen with the W7A-NTF2 mutant (28), in which the avidity for FG Nups is impaired. Finally, during promiscuous binding of NTF2 in the presence of Kapβ1, we found that Kapβ1 retention within Nsp1p was long-lived and prevented brush collapse when NTF2 bound. This promoted faster NTF2 dissociation kinetics and supports the proposal (21,22) that Kapβ1 contributes together with FG Nups to generate the NPC barrier function. Thus, the amount of bound Kapβ1 could potentially influence both NPC permeability and rapid selective transport.

MATERIALS AND METHODS

Cloning and expression of recombinant proteins

Wild-type NTF2

The full-length wild-type rat NTF2 coding sequence (29) was cloned into the *NdeI* and *XhoI* sites of the T7 expression vector pET15b (Novagen), with the addition of an N-terminal His_6-tag. The construct was transformed into *Escherichia coli* strain BL21(DE3) CodonPlus RIL, expressed, and purified using NiNTA agarose and gel filtration (Superdex S-75; GE Healthcare) as previously described (29).

W7A-NTF2

PCR-based, site-specific mutagenesis was used to obtain the rat W7A mutant of NTF2 as previously described (30,31). The sequence was cloned into the T7 expression vector pET15b, expressed in *E. coli* BL21(DE3), and purified using ion-exchange chromatography and gel filtration as previously described (29).

Nsp1p-5FF and Nsp1p-12FF

Two yeast Nsp1p FG-fragments, Nsp1p-5FF (residues 262–359; 1× FG, 4× FSFG) and Nsp1p-12FF (residues 262–492; 1× FG, 11× FSFG), were cloned via *NcoI* and *HindIII* sites into a modified pET30a vector (Novagen) whose thrombin protease recognition site was changed for TEV protease and Cys-Cys-Trp was added after its initiator Met codon. The additional Cys residues facilitated coupling to the gold SPR sensor surface, whereas the Trp residue enabled us to determine the protein concentration by measuring the optical density at 280 nm. To express proteins in BL21(DE3) CodonPlus RIL, cells were grown at 37°C in 2× TY media to OD_{600} 0.6 and induced with 1 mM isopropyl β-D-1-thiogalactopyranoside overnight at 25°C. The cells were lysed in 50 mM Tris-HCl pH 8.0/1 mM EGTA/25% (w/v) sucrose/8 M urea by using an EmulsiFlex C3 homogenizer (Avestin) at a pressure of 15,000 psi in the presence of 1 mM PMSF. Proteins were purified under native conditions using NiNTA agarose (Qiagen) according to the manufacturer's instructions, and then by size-exclusion chromatography on a Superdex S-75 26/60pg column (GE Healthcare) in 20 mM Tris-HCl pH 8.0/1 mM dithiothreitol/50 mM NaCl.

Kapβ1

Full-length human Kapβ1 was cloned, expressed, and purified as previously described (21). The functionality of these proteins is conserved across species (32).

Protein quality (see Fig. S1 in the Supporting Material) was assessed by SDS-PAGE and concentrations were measured by absorption at 280 nm. Protein extinction coefficients were obtained using the ProtParam program (http://web.expasy.org/protparam/).

SPR measurements

A four-flow cell Biacore instrument (T100; GE Healthcare) was used to measure SPR at 25°C in PBS, pH 7.2 (GIBCO by Life Technologies), as previously detailed (22). Briefly, each experiment included two reference cells and two sample cells. Reference cells were prepared by covalently grafting $C_{17}H_{36}O_4S$ (hydroxyl-terminated tri(ethylene glycol) undecane thiol, HS-(CH_2)-$(OCH_2CH_2)_3$-OH; Nanoscience) onto a gold sensor surface via thiol binding. Sample cells were prepared by covalently grafting cysteine-modified Nsp1p fragments onto each respective gold sensor surface followed by $C_{17}H_{36}O_4S$ to further passivate any exposed gold. Different grafting distances were obtained by changing the incubation time for the Nsp1p fragments. A 1% (w/v) bovine serum albumin (BSA; Sigma-Aldrich) solution was prepared in PBS (pH 7.2). Before experiments were conducted, Kapβ1, NTF2, W7A-NTF2, and both Nsp1p fragments were dialyzed into PBS buffer (pH 7.2). All protein and reagent solutions were centrifuged for 15 min at 16,000 × g to remove particles and gas bubbles. Buffer solutions were filtered (0.22 μm) and degassed before use. Postexperiment checks ensured that covalent binding of Kaps to the underlying gold surface did not occur (Fig. S2). In all cases, layer height was measured after a dissociation phase of 480 s due to technical limitations that prevented the simultaneous injection of BSA with the respective NTR. Therefore, the BSA signal obtained for the bound material $R_{bound,i}$ underestimated the height at equilibrium binding $R_{eq,i}$ (Fig. S3). The total number of experiments, N, was as follows: Kapβ1 on Nsp1p-12FF ($N = 8$), NTF2 on Nsp1p-5FF ($N = 11$), NTF2 on Nsp1p-12FF ($N = 15$), and NTF2/Kapβ1 on Nsp1p-12FF ($N = 5$).

Multivalent binding analysis

A model that calculates a discrete distribution of kinetic states ($k_{on,i}$,$k_{off,i}$) (26) was used to fit the measured SPR sensorgrams for Kapβ1 as previously described (21). For NTF2, we used a simplified two-dimensional lattice of 5 × 5 nm^2 NTF2-binding spots to describe the FG-repeat-containing surface, taking the average Stokes radius of an NTF2-dimer as 2.5 nm (2) (Supporting Material and Fig. S4). In brief, a set of 36 × 36 ($k_{on,i}$, $k_{off,i}$) pairs was populated and their fractional abundance was depicted as color intensity in k_{on}-versus-KD and k_{off}-versus-KD interaction maps averaged over ~10 individual sensorgrams. Calculations and visualizations were obtained using MATLAB (The MathWorks, Natick, MA) and Python.

RESULTS

Close-packed Nsp1p FG domains form a molecular brush

SPR measures the binding and release of analytes from surface-tethered ligands. We previously extended this technique to show that noninteracting BSA molecules could be used to determine the average height h of a surface layer

(22), and validated the BSA-SPR measurements by using atomic force microscopy (AFM) (33). Briefly, the magnitude of the BSA-SPR signal (in terms of resonance units (RU)) gives a measure of h because thicker layers give smaller signals than thinner layers. Details of the BSA-SPR method, including calculations of the grafting distance, g, for immobilized proteins from the SPR response (using the relation 1300 RU = 1 ng/mm^2), can be found in previous publications (21,22,33).

Two different Nsp1p fragments, Nsp1p-5FF and Nsp1p-12FF, were used in the SPR experiments. Both constructs contain N-terminal 2× Cys-, His$_6$-, and S-tags, and have equally spaced FG repeats separated by hydrophilic linker regions. Dynamic light scattering (DLS) gave their hydrodynamic radii (r_h) as 4.4 ± 1.0 nm for Nsp1p-5FF and 4.3 ± 1.3 nm for Nsp1p-12FF, although r_h of Nsp1p-5FF may have been slightly overestimated due to polydispersity (Supporting Material). As shown in Fig. 1, surface-tethered Nsp1p layers exhibited a steep increase in layer height, indicating that close packing ($g < r_h$) resulted in molecular brush formation (34). The average brush heights were h_{5FF} = 11.0 ± 1.2 nm, which was smaller than h_{12FF} = 15.7 ± 2.7 nm. Importantly, the average FG repeat volume densities were 0.058 FG/nm^3 (Nsp1p-5FF) and 0.062 FG/nm^3 (Nsp1p-12FF), respectively, reproducing the anticipated FG repeat density within the yeast NPC (0.08 FG/nm^3) (35).

Binding of Kapβ1, NTF2, and W7A-NTF2 to Nsp1p FG brushes

Fig. 2 *A* shows the close-packed Nsp1p-12FF brush height, h_i, normalized by its initial height, h_0, measured after each consecutive injection, i, of Kapβ1. Brush collapse was observed below 100 nM Kapβ1, followed by a 50% layer extension in 10 μM Kapβ1 that reached a height of ~24 nm. This height indicated that the Nsp1p brush was fully occupied by approximately three Kapβ1 layers (Supporting Material) based on the ~10 nm hydrodynamic diameter of Kapβ1 and a bound surface density, $\rho_{\mathrm{Kap}\beta 1}$, of 3330 Da/nm^2 (where one Kapβ1 layer = 1000 Da/nm^2) (22) (Fig. 2 *B*). This was comparable to how Kapβ1 binds the FxFG domains of Nup214, Nup62, and Nup153 (21).

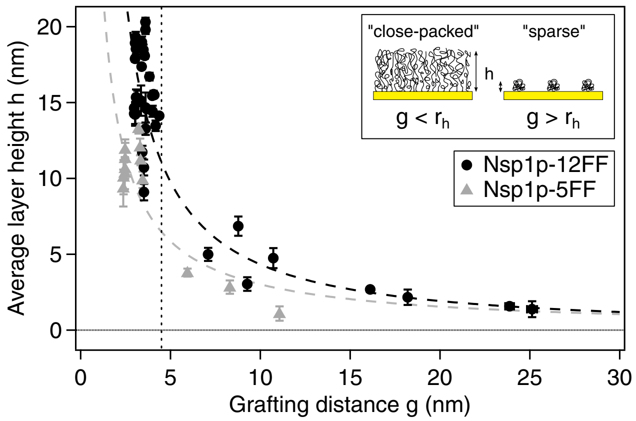

FIGURE 1 Average layer height, h, as a function of grafting distance, g, for both Nsp1p FG domain fragments. The vertical dashed line corresponds to their hydrodynamic radii, r_h, of 4.5 nm. Flory-Huggins fits predict polyelectrolyte brush behavior. Inset: cartoon description of a molecular brush for $g < r_h$ (close-packed) and mushrooms for $g > r_h$ (sparse). To see this figure in color, go online.

We then compared NTF2's interaction with the Nsp1p-12FF brush and its interaction with Kapβ1, using as a negative control the NTF2 W7A mutant (W7A-NTF2), in which FG Nup binding is impaired (28). Fig. 2 *C* shows that the change in layer height was negligible for both proteins at low concentrations. For wild-type NTF2, a decrease in layer height started at an NTF2 concentration of ~1 μM, reached a ~12% (2 nm) reduction at physiological concentrations (~20 μM) (36), and reached an overall reduction of 15% at the highest concentration tested (~270 μM). No change in layer height was observed with the W7A mutant, even at extremely high concentrations (up to ~300 μM), consistent with previous studies showing that a reduced avidity of the W7A mutant for Nsp1p impaired NTF2-mediated nuclear import of RanGDP (28,37). Whereas up to 1400 Da/nm^2 or approximately one layer of wild-type NTF2 was bound (where one layer of NTF2 = 1342 Da/nm^2), less than 100 Da/nm^2 of W7A-NTF2 was bound (equivalent to ~0.05 layers) at the highest injected bulk concentration (Fig. 2 *D*).

Binding avidity of Kapβ1, NTF2 and W7A-NTF2 to Nsp1p FG brushes

Fig. 3 shows the equilibrium binding responses of Kapβ1, NTF2, and W7A-NTF2 to Nsp1p-12FF. Because in each case single isotherm fits proved suboptimal (indicating there was multivalent binding), we analyzed these data by using a two-component Langmuir isotherm. For Kapβ1, a high-avidity species with KD_1 = 336 ± 63 nM represented tight binding at high FG repeat density in close-packed Nsp1p FG brushes, whereas moderate binding at KD_2 = 5.6 ± 2.0 μM was consistent with reduced binding due to preoccupancy of Kapβ1 and a limited access to FG repeats within the layer (21). NTF2 gave dissociation constants of KD_1 = 2.1 ± 0.5 μM and KD_2 = 114 ± 23 μM, which were similar for Nsp1p-5FF and Nsp1p-12FF (Fig. S5). KD_2 indicated that a nonnegligible fraction of NTF2 bound to the Nsp1p FG domains much more weakly than the known primary physiological interaction (28,36). In comparison, a marked reduction in binding was observed for W7A-NTF2 that had KD_1 = 18.8 ± 3.0 μM and KD_2 = 356 ± 44 μM. In spite of KD_1 being about an order of magnitude weaker than wild-type NTF2, the remaining low avidity given by KD_2 for W7A-NTF2 indicated the existence of less specific FG binding sites on NTF2, as predicted by NMR (38) and computational studies (39,40).

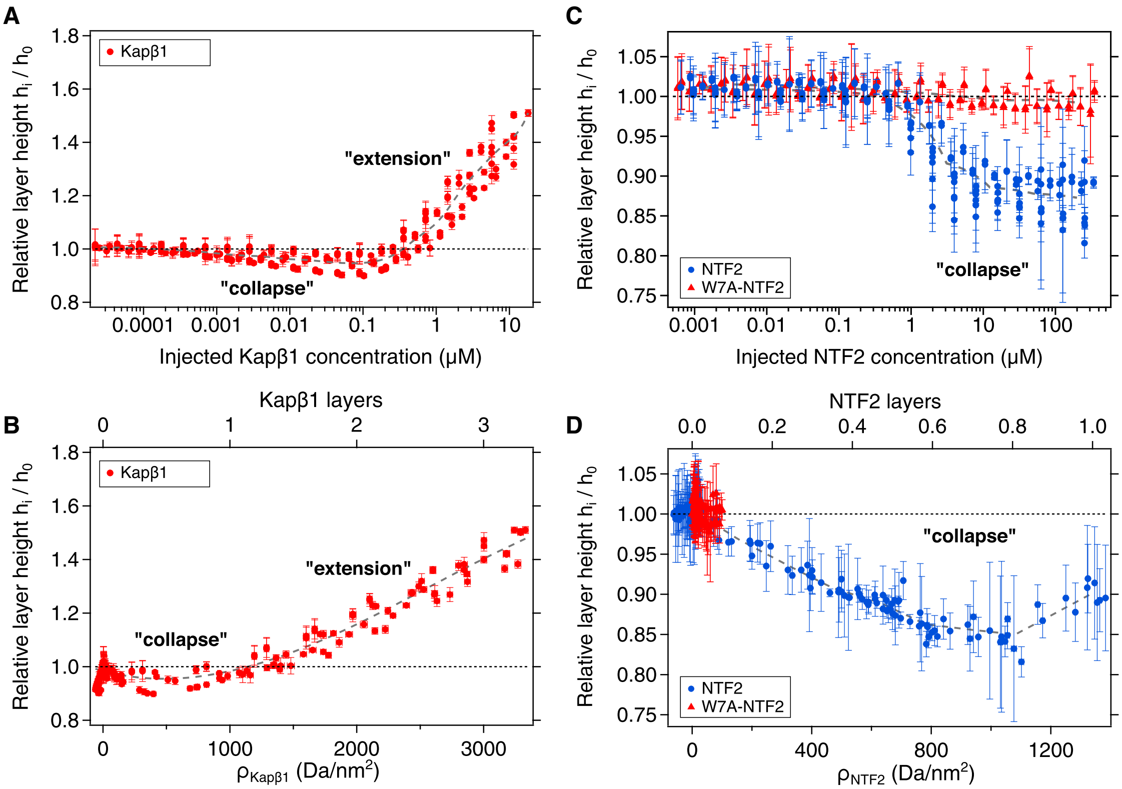

FIGURE 2 Conformational response of close-packed Nsp1p-12FF layers upon binding Kapβ1, NTF2, and W7A-NTF2. (*A–D*) The relative layer height is shown as a function of (*A*) injected Kapβ1 bulk concentration, (*B*) surface density and equivalent number of bound Kapβ1 layers, (*C*) injected NTF2 or W7A-NTF2 bulk concentration, and (*D*) NTF2 or W7A-NTF2 surface density and equivalent number of bound layers. Collapse was not observed for W7A-NTF2 binding. Error bars are ± SD. Dashed gray lines represent a sliding average. To see this figure in color, go online.

Analyses of multivalent binding kinetics to Nsp1p FG brushes

Although an equilibrium binding analysis provides thermodynamic information (e.g., on the stability of the NTR-Nsp1p complex), the temporal transition between bound and unbound NTR forms depends on the kinetic on- and off-rates (k_{on} and k_{off}, respectively). Therefore, we applied the method of Svitel et al. (26) to identify fast- and slow-binding populations of each respective NTR, as was previously done for Kapβ1 (21). In this manner, we could obtain a more resolved distribution of KDs by knowing k_{on} and k_{off}.

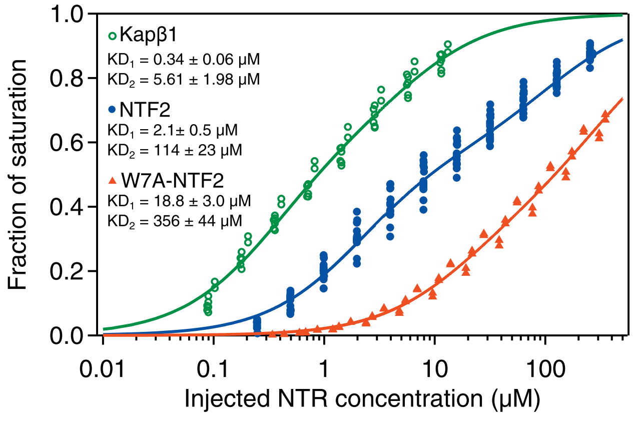

FIGURE 3 Semi-log plot showing the equilibrium binding of Kapβ1 (*open circles*), NTF2 (*solid circles*), and W7A-NTF2 (*triangles*) to Nsp1p-12FF brushes. The data were normalized by the maximum binding capacity (fraction of saturation) and are shown as a function of injected bulk NTR concentration. Solid lines represent the average two-component Langmuir isotherm for Kapβ1, NTF2, and W7A-NTF2, respectively. To see this figure in color, go online.

Fig. 4 *A* shows that Kapβ1 binding to Nsp1p-12FF features a broad distribution of affinities ranging from nanomolars to micromolars. Except for the peak at ~20 nM, the KDs at ~150 nM and ~3–5 μM were in good agreement with the KDs from the equilibrium binding analysis (Fig. 3). At low Kapβ1 concentrations, a high-avidity slow phase (○) commenced at $k_{on} = 1.2 \times 10^4$ s^{-1}M^{-1}, $k_{off} = 1.3 \times 10^{-5}$ s^{-1}, resulting from a long-lived half-life of $t_{1/2} \approx 15$ h (where $t_{1/2} = \ln(2)/k_{off}$). Increasing the concentration toward 10 μM Kapβ1 led to a steady reduction in k_{on} to ~60 s^{-1}M^{-1} (Δ), giving rise to lower-avidity interactions (increasing KD) that coincided with the emergence of a low-avidity fast phase (*) having a fast k_{on} (~1.6×10^5 s^{-1}M^{-1}) and a fast k_{off} (0.1–1.6 s^{-1}), where now $t_{1/2}$ = 430 ms to 7 s. These results were consistent with Kapβ1 binding to human FG domains observed previously (21), and were indicative of an overall reduction in avidity resulting from 1) a reduction of available FG repeats, 2) poor penetration due to

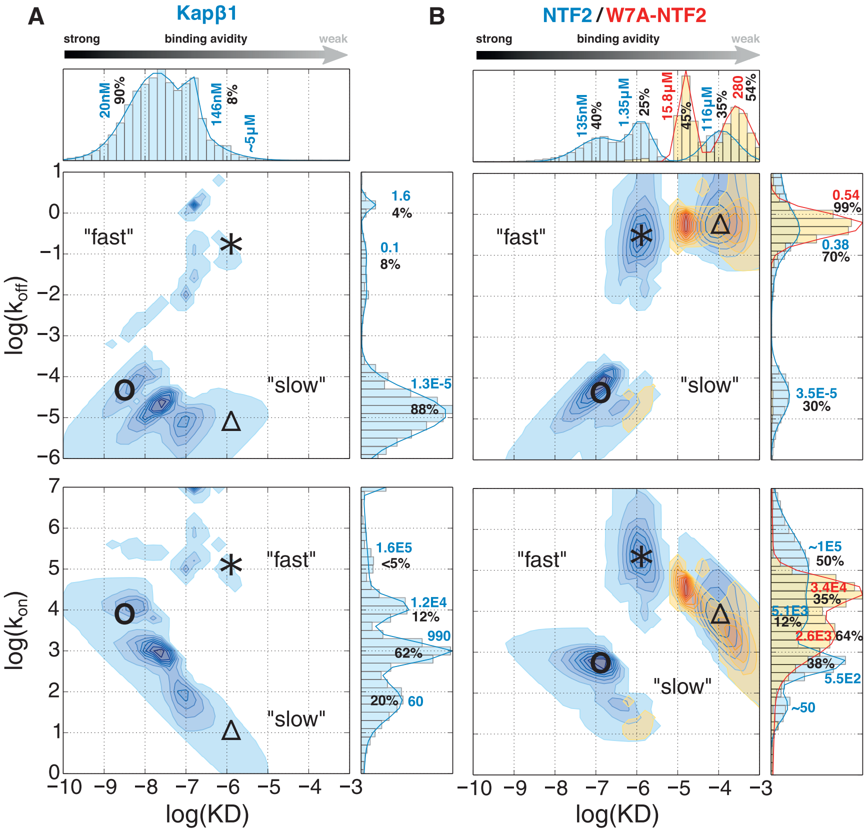

FIGURE 4 (*A* and *B*) Multivalent kinetic analysis of (*A*) Kapβ1 and (*B*) NTF2/W7A-NTF2 binding to Nsp1p-12FF brushes. Two-dimensional interaction maps of kinetic on- and off-rates (k_{on} and k_{off}, respectively) are shown with their derived equilibrium binding constant, KD. The fractional abundance of different kinetic states is indicated by the color intensity and the sum over all values in a given axis is shown as accompanying histograms (*top and right panels*). Each distribution is given in percent of the total sum and their main values are in bold. For Kapβ1, the different kinetic species are labeled with ○ (high-avidity slow phase), * (low-avidity fast phase), and Δ (low-avidity slow phase). For NTF2, the different kinetic species are labeled with ○ (high-avidity slow phase), * (mid-avidity fast phase), and Δ (low-avidity fast phase). Values corresponding to W7A-NTF2 are depicted in red. Units are s^{-1} and $s^{-1}M^{-1}$ for k_{off} and k_{on}, respectively.

Kapβ1 occupancy and crowding, 3) a reduced mobility of flexible FG chains due to Kapβ1 binding, and 4) steric repulsion due to FG chain extension. In this respect, the coexistence of both slow (low k_{off}) and fast phases (high k_{off}) at μM Kapβ1 concentrations indicated that the quantity and/or accessibility of the FG repeats was reduced as Kapβ1 accumulated in the layer.

Fig. 4 *B* summarizes the distribution of k_{on} and k_{off} obtained for the binding of NTF2 and W7A-NTF2 to Nsp1p-12FF. For NTF2, the obtained KDs gave distinct peaks at ~100 nM, ~1 μM, and ~100 μM. Overall, we identified three distinctive kinetic species: 1) a high-avidity slow phase (○) with low k_{on} (~500 $s^{-1}M^{-1}$), low k_{off} (~3.5 × 10^{-5} s^{-1}), and long half-life of $t_{1/2} \approx 5.5$ h; 2) a mid-avidity fast phase (*) with high k_{on} (~10^5 $s^{-1}M^{-1}$), high k_{off} (between 0.3–10 s^{-1}), and short $t_{1/2}$ of ~70 ms to 2 s; and 3) a low-avidity fast phase (Δ) consisting of a reduced k_{on} (~5100 $s^{-1}M^{-1}$) and a similar high k_{off} compared with the mid-avidity fast phase. The apparent bimodal distribution of k_{off} was consistent with the presence of two major complexes with different stabilities. Although high micromolar-to-millimolar affinities are often considered as nonspecific, they are relevant for NTRs binding to individual FG repeats during transit through the NPC transport channel because of their high off-rates (19). Except for the low KD range peaking around ~100 nM, the KD distribution obtained from the multivalent kinetic analysis was in good agreement with the KDs from the equilibrium binding analysis (Fig. 3). Overall, the Nsp1p-5FF and Nsp1p-12FF FG domain constructs gave very similar results (Fig. S6).

By comparison, a substantially weaker complex formed during W7A-NTF2 binding to Nsp1p FG repeats, as underscored by the absence of a high-avidity slow phase (Fig. 4 *B*). This indicated binding affinities of approximately 16 μM and 300 μM, in good agreement with the Langmuir isotherm analysis (Fig. 3). Hence, W7A-NTF2 still bound to the FG domains via a number of other putative sites (38,40), although its primary FG repeat binding site at Trp7 is impaired. Conversely, this confirmed that Trp7 is required for the high-avidity, slow-phase binding of wild-type NTF2 that leads to the collapse of close-packed Nsp1p FG domains (Fig. 2 *C*).

Promiscuous binding of Kapβ1 and NTF2 to Nsp1p FG brushes

We then investigated how binding promiscuity would affect Kapβ1 and NTF2 binding. Generally, resolving how two different analytes interact simultaneously with surface-tethered ligands is not straightforward in SPR. However, in these circumstances, it was permissible to analyze this because the majority of Kapβ1 molecules that bind and occupy Nsp1p were far longer lived than NTF2 (Fig. 4). These effects are readily visible in the representative data

shown in Fig. 5. For clarity, one measurement contained the binding of up to ~15 μM Kapβ1 followed by increasing titrations of NTF2 (Fig. 5 *A*). Another measurement contained the binding of up to ~15 μM Kapβ1 followed by blank injections (i.e., PBS buffer; Fig. 5 *B*).

After eluting for 2230 s past the final Kapβ1 injection, ~2.5 layers or 80% of Kapβ1 remained bound in the Nsp1p brush that had extended by 40% over its initial height (Fig. 5 *C*). Surprisingly, both NTF2 (Fig. 5 *A*) and blank (Fig. 5 *B*) injections elicited the same height change from this Kapβ1-preloaded brush, which reduced to a 20% extension at the highest NTF2 concentration (i.e., 270 μM; Fig. S7). This indicated that NTF2 binding did not significantly impact the structural integrity of Nsp1p in the presence of strongly bound Kapβ1, which clearly had very slow off-rates. Indeed, if NTF2 binding facilitated Kapβ1 dissociation (washing out of bound Kapβ1), one would anticipate a more marked reduction in layer height (Fig. 2 *C*). We then subtracted the intrinsic slow phase of Kapβ1 (Fig. 5 *B*) from the combined Kapβ1/NTF2 SPR signal (Fig. 5 *A*) to decouple and isolate the signal of promiscuously bound NTF2 (Fig. S8).

Subsequent multivalent analyses revealed that the difference between promiscuous NTF2 binding in the presence of Kapβ1 compared with NTF2 binding pristine Nsp1p brushes was significant. As shown in Fig. 6, NTF2 binding

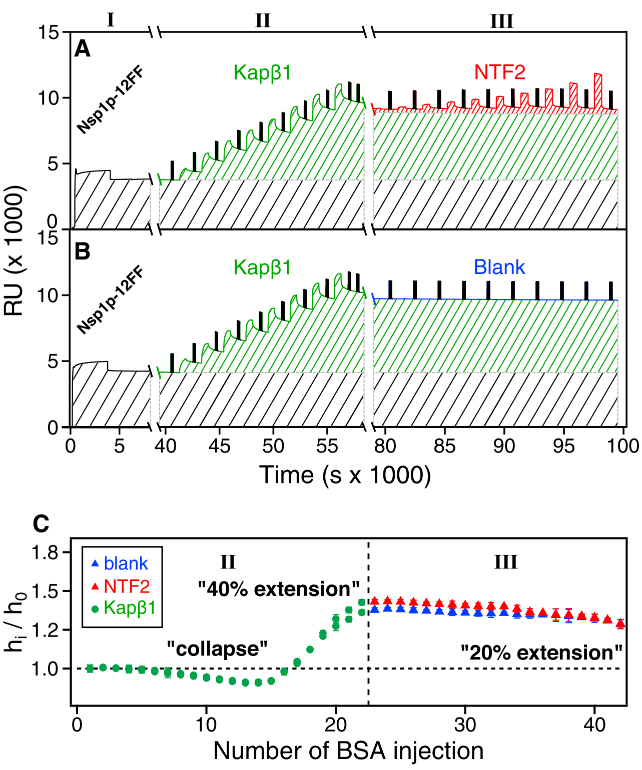

FIGURE 5 (*A* and *B*) Representative data showing the SPR response of (*A*) NTF2 binding (*red shaded area*) and (*B*) blank PBS (*blue shaded area*) injections to Kapβ1-preloaded (*green shaded area*) Nsp1p-12FF brushes (*black shaded area*), respectively. For clarity, the black spikes correspond to BSA injections. In both cases, Kapβ1 binding to Nsp1p-12FF is long-lived with a considerable occupancy. In comparison, NTF2 binding to Nsp1p-12FF is short-lived with a far lower occupancy. (*C*) Corresponding height changes in a Kapβ1-preloaded Nsp1p-12FF layer after NTF2 injections (*vertical dashed line*). The layer transitions from a 40% extension at 15 μM Kapβ1 to a 20% extension in 270 μM NTF2. Note the similarity in layer height when blanks (i.e., PBS) are injected.

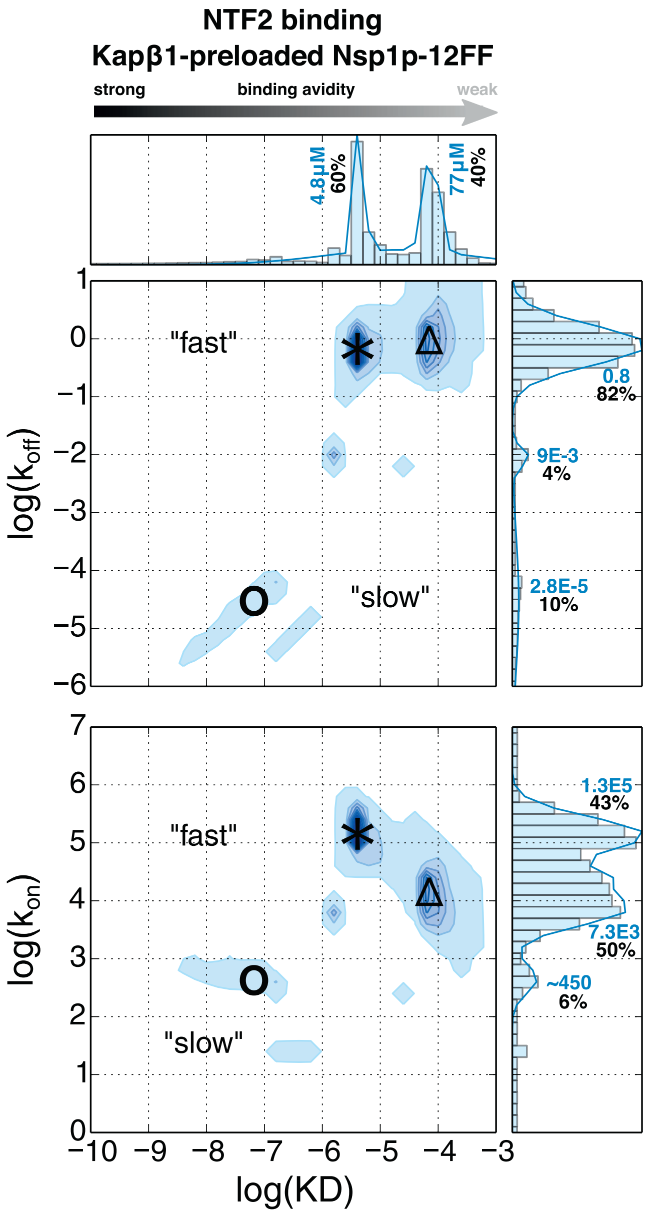

FIGURE 6 Multivalent kinetic analysis of NTF2 binding close-packed Nsp1p FG domains preloaded with Kapβ1. Two-dimensional interaction maps of kinetic on- and off-rates (k_{on} and k_{off}, respectively) are shown in relation to the equilibrium binding constant KD. The fractional abundance of different kinetic states is indicated by the color intensity and the sum over all values in a given axis is shown as accompanying histograms (*top and right panels*). Different kinetic species are labeled with ○ (high-affinity slow phase), * (mid-affinity fast phase), and Δ (low-affinity fast phase). Each distribution is given in percent of the total sum and their main values are depicted in bold. Units are s^{-1} and $s^{-1}M^{-1}$ for k_{off} and k_{on}, respectively. To see this figure in color, go online.

avidity was dominated by weak KDs at 4.8 μM and 77 μM, where 80% of the bound fraction exhibited fast k_{off} (i.e., 1 s^{-1}; $t_{1/2} = 70$ ms; see Fig. S9 for equilibrium binding analyses). This was consistent with a lack of significant competition between the already bound Kapβ1 and the added NTF2. Hence, an overall trend toward faster and more transient interactions of NTF2 was observed when Kapβ1 was present in the Nsp1p brush. This corresponded to 0.06 layers of NTF2 at the highest injected concentration of 270 μM.

DISCUSSION

Nsp1p FG domains form a molecular brush

FG domain morphology and its response to binding are strongly dependent on surface tethering (41) because this imposes a surface boundary that limits NTR occupancy (21). Due to lateral crowding, entropic effects dominate over, but do not preclude, competing enthalpic interactions between chains (i.e., cohesion), resulting in Nsp1p forming a molecular brush. Importantly, the close agreement between the FG repeat density (~0.06 FG/nm^3) obtained in this study and that obtained in yeast NPCs (0.08 FG/nm^3) (35) makes it an attractive in vitro system in which to study the functional properties of FG Nups when they are binding different NTRs.

NTF2 binding leads to Nsp1p brush collapse and Kapβ1 drives its expansion

Our results demonstrated that NTF2 and Kapβ1 binding to FG regions of Nsp1p influenced the brushes very differently. Surprisingly, the Nsp1p brush exhibited collapse at even the highest NTF2 concentrations used. Within the physiological range (~20 μM NTF2), the collapse was ~12% of the initial layer height, with the bound content corresponding to effectively one monolayer of NTF2. In comparison, Kapβ1 binding was characterized by a nonmonotonic response that collapsed the Nsp1p brush at low nanomolar concentrations (7), followed by a self-healing extension (22) at physiological (μM) concentrations. This was due to an increasing occupancy of Kapβ1, which formed multilayers within the brush, and was consistent with SPR measurements of Kapβ1 binding to Nup214, Nup62, and Nup153 (21).

These data show a size-dependent effect that differentiated NTF2 (small) from Kapβ1 (large), and support the theory of Opferman et al. (42,43), which predicts that binding-induced conformational changes in polymer brushes depend on the nanoparticle size and the interaction energy with the polymer. Thus, changes in brush height originate from competition between the binding energy of nanoparticles to the polymer, favoring collapse, and the confinement entropy of the polymers, promoting extension. Although Kapβ1 showed a higher avidity for FG repeats than NTF2, its binding at physiological concentrations favored layer extension because of its relatively large volume, which impacts the entropy of the FG domains. Because NTF2 is smaller, its binding favors collapse over extension, although the latter may be possible at higher (but nonphysiological) concentrations. By contrast, W7A-NTF2 did not collapse the brush because it only bound very weakly to Nsp1p.

Our results are consistent with measurements of Kap95p (yeast importinβ) binding to Nsp1p residues 2–601 in layers with comparable surface grafting densities (~4 nm) (44). The two-component KD we obtained by SPR (340 nM and 5.6 μM) was indistinguishable from the KD values (320 nM and 5.3 μM) obtained by ellipsometry (44). Notwithstanding methodological differences, the SPR-measured height increase was also comparable to the ~4 nm Nsp1p layer extension seen with 5 μM Kap95p using a quartz crystal microbalance with dissipation (44). Coincidentally, the average FG repeat concentration of 106 ± 18 mM (i.e., 0.064 FG repeats/nm^2) reported by Eisele et al. (44) was equivalent to the FG repeat density obtained here. Indeed, the transition from brush collapse into extension we found at 0.2 μM Kapβ1 (Fig. 2 *A*) may explain why AFM did not detect Nsp1p collapse at similar concentrations of Kap95p. On a more technical note, our SPR method is limited to static height measurements and cannot capture dynamic reversible collapse events of single FG Nups, such as those obtained by single-molecule fluorescence (45).

Kinetic analysis of multivalent binding

Understanding how NTF2 and Kapβ1 bind Nsp1p separately provides benchmarks for the avidity that is manifest from multivalent interactions with proximal FG domains. Overall, both NTF2 and Kapβ1 formed more than one complex with the Nsp1p FG domains. This was evident from the existence of multiple KDs, as obtained from equilibrium binding analyses and the distribution of k_{on} and k_{off} obtained from multivalent kinetic analyses. The structural basis of this behavior is likely complicated, but can be rationalized given that a single Nsp1p chain can bind multiple copies of the same NTR (one to many) or several FG domains can bind simultaneously to a single NTR (many to one), or a combination of both characteristics could occur. This is consistent with the behavior of intrinsically disordered proteins (25).

The kinetics of Kapβ1 binding to Nsp1p was similar to that observed for its binding to human Nup214, Nup62, Nup98, and Nup153 (21). This was characterized by ~90% of bound Kapβ1 exhibiting stronger and longer complex lifetimes (low k_{off}) accompanied by a minority exhibiting high off-rates associated with binding at the Nsp1p periphery (Fig. 4). In contrast, NTF2 binding was more transient, with 70% of bound molecules showing fast off-rates and 99% of W7A-NTF2 being in this fast regime. Except for the high-avidity complex formed at KD = 135 nM, the

~1 to 2 μM and ~100 μM KDs obtained for NTF2 from both equilibrium and kinetic analyses were consistent with previous single-value estimates (28,36). Because NTF2 has fewer FG binding sites and is smaller in size than Kapβ1, its multivalent binding kinetics may be dominated less by in-layer crowding and more by local structural effects, especially since NTF2 occupancy only reached one layer in the Nsp1p brush even at the highest titrates (Fig. 2 *D*). Its low- and high-avidity modes may result from the occupation of one or two FG binding sites on the NTF2 dimer, respectively. Alternatively, NTF2 could bind two FG repeats on a single Nsp1p chain or to single FG repeats on two different Nsp1p chains. We speculate that the latter interaction would be more favored energetically, since the former would more considerably restrict the Nsp1p conformation. Irrespective of the precise mechanism involved, impairing the primary FG interaction sites on the W7A mutant impacted both interactions.

Promiscuous binding of NTF2 to Nsp1p in the presence of Kapβ1

Preloading Nsp1p brushes with Kapβ1 had a dramatic influence on the binding of NTF2. Binding Kapβ1 to Nsp1p should reduce its flexibility (so Nsp1p becomes increasingly rigid (7,44)) and also reduce the availability of free FG repeats. Consequently, the avidity of NTF2 would be weakened by the extent to which Kapβ1 is bound. Because Kapβ1 binds more strongly to Nsp1p than NTF2, its occupancy is higher, forcing the layer to extend and making it harder for NTF2 molecules to penetrate the Kapβ1-dominated volume. Under these conditions, kinetic analysis indicated that the two stronger, specific interaction modes identified with NTF2 alone were altered in a manner similar to that observed when the W7A mutant bound to a pristine Nsp1p brush. Here, the strongest mode was essentially eliminated, whereas the avidity of the weaker mode was reduced and the weakest (probably nonspecific) binding was not altered greatly. Reduced Nsp1p chain flexibility may increase the entropic cost of binding two Nsp1p chains to a single NTF2 dimer and thus inhibit formation of the strongest binding mode. Similarly, the entropic penalty associated with binding a single chain would also increase, resulting in decreased avidity and hence an increase in the bound NTF2 population with high off-rates (i.e., 80%; Fig. 6).

Kapβ1 contributes to the NPC barrier function and promotes fast NTF2 kinetics

Recently, it was proposed that Kapβ1 is an integral, bona fide constituent of the NPC barrier, which is often assigned to the FG Nups alone, and that Kapβ1 contributes to modulating both mechanistic and kinetic aspects of NPC barrier functionality (21). Here, the stronger and longer-lived FG domain-binding interactions exhibited by Kapβ1 compared with those of NTF2 provide support for such a Kap-centric barrier mechanism (21,22). In this context, promiscuous binding of Kapβ1 may be essential to maintain NPC barrier function by increasing the rigidity of the FG domain layer (7,44) to increase the barrier against molecules that bind nonspecifically (23,46). Indeed, studies show that the immobile fraction of Kapβ1 (~100 molecules/pore) is substantially larger than that of NTF2 (~6 molecules/pore) (20).

As illustrated in Fig. 7, the presence of slow-phase Kapβ1 would hinder and limit how far NTF2 penetrates into the FG layer, thereby counterbalancing NTF2-mediated FG domain collapse. Accordingly, the fast interaction kinetics (high k_{off}) of NTF2 could promote selective diffusion along the peripheral regions of the engorged FG domains in a manner that is contiguous with the fast Kapβ1 phase (21), such as by a reduction of dimensionality (23,47). Indeed, both NTRs appear to traverse NPCs simultaneously and with similar dwell times of ~5 ms (48,49). Consistent with

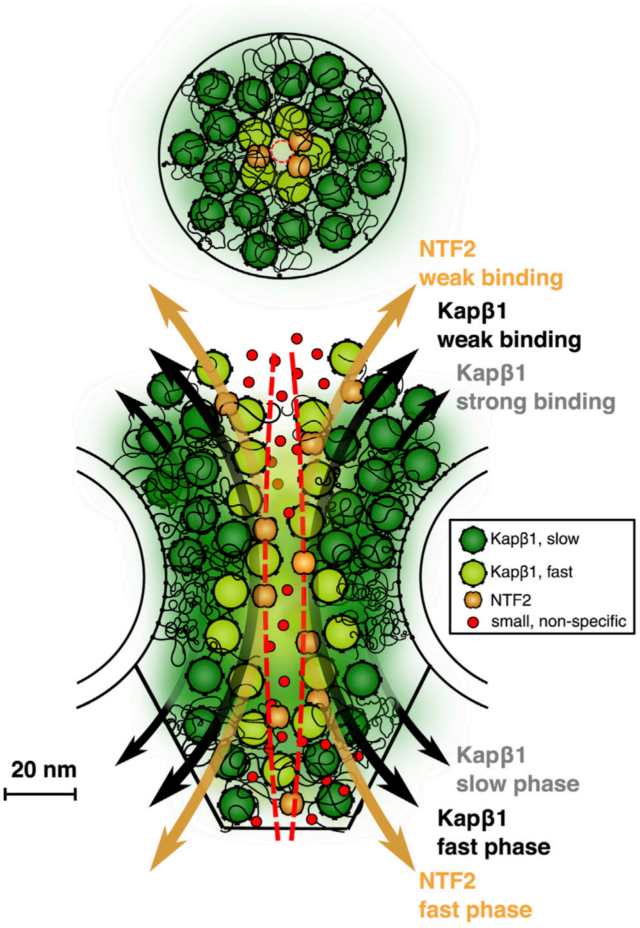

FIGURE 7 Kap-centric barrier model showing how different NTRs may share contiguous spatial and temporal routes through the NPC. Strongly bound Kapβ1 molecules (slow) occupy the FG Nups and form integral constituents of the barrier mechanism. This crowding promotes the facilitated diffusion of NTF2 and a smaller fraction of Kapβ1 (fast) through a central conduit bearing a reduced density of FG repeats. To see this figure in color, go online.

the Kap-centric model, in vitro nuclear protein import assays show increased transport rates with increasing Kapβ1 concentrations (24). We further speculate that decreasing the effective Kapβ1 concentration or occupancy at the NPC would generate a less effective barrier (i.e., more open, less selective) due to NTF2-mediated FG domain collapse.

A formidable challenge lies in decoupling the diverse pathways that converge on NPCs, constituting the main nucleocytoplasmic transport hub (50). Clearly, the pore channel is crowded (20), and it is essential to know the effective local concentrations (51) of each transport receptor in and around the NPC. It is also crucial to establish how the loading of Kapα and specific cargoes influences Kapβ binding, and the extent to which different NTRs bind preferentially to different FG Nups. In terms of binding promiscuity, this could demarcate not only spatial pathways (52) but also temporal ones. Irrespective of the precise mechanisms involved, promiscuous binding and the influence of Kapβ1 binding on the off-rate of other NTRs clearly make contributions that one should take into account when formulating precise models of nucleocytoplasmic transport.

CONCLUSIONS

To our knowledge, these results demonstrate for the first time that promiscuous binding of NTRs to FG Nups should influence nucleocytoplasmic transport. This depends on the concentration, size, and binding strength of each NTR. Indeed, some form of hierarchy may exist between different NTRs such that their relative concentrations may impact NPC barrier function. This interpretation departs from the conventional view that the FG Nups alone form the NPC permeability barrier. Rather, we propose that concentrating NTRs in the NPC transport channel also contributes to generating the crowding-based selective barrier function of the pore.

SUPPORTING MATERIAL

Supporting Materials and Methods and nine figures are available at http://www.biophysj.org/biophysj/supplemental/S0006-3495(14)04820-6.

ACKNOWLEDGMENTS

We thank A. Zilman for stimulating discussions.

This work was supported by the Swiss National Science Foundation (R.Y.H.L.), the Biozentrum (R.Y.H.L.), and the Swiss Nanoscience Institute (R.Y.H.L.). Further support was provided by Medical Research Council grant U105178939 (M.S.) and Wellcome Trust Programme grant 080522 (M.S.).

REFERENCES

1. Beck, M., F. Förster, ..., O. Medalia. 2004. Nuclear pore complex structure and dynamics revealed by cryoelectron tomography. *Science.* 306:1387–1390.
2. Ribbeck, K., and D. Görlich. 2001. Kinetic analysis of translocation through nuclear pore complexes. *EMBO J.* 20:1320–1330.
3. Elad, N., T. Maimon, ..., O. Medalia. 2009. Structural analysis of the nuclear pore complex by integrated approaches. *Curr. Opin. Struct. Biol.* 19:226–232.
4. Paine, P. L., L. C. Moore, and S. B. Horowitz. 1975. Nuclear envelope permeability. *Nature.* 254:109–114.
5. Keminer, O., and R. Peters. 1999. Permeability of single nuclear pores. *Biophys. J.* 77:217–228.
6. Rout, M. P., J. D. Aitchison, ..., B. T. Chait. 2000. The yeast nuclear pore complex: composition, architecture, and transport mechanism. *J. Cell Biol.* 148:635–651.
7. Lim, R. Y. H., B. Fahrenkrog, ..., U. Aebi. 2007. Nanomechanical basis of selective gating by the nuclear pore complex. *Science.* 318:640–643.
8. Lim, R. Y. H., N. P. Huang, ..., U. Aebi. 2006. Flexible phenylalanine-glycine nucleoporins as entropic barriers to nucleocytoplasmic transport. *Proc. Natl. Acad. Sci. USA.* 103:9512–9517.
9. Frey, S., and D. Görlich. 2007. A saturated FG-repeat hydrogel can reproduce the permeability properties of nuclear pore complexes. *Cell.* 130:512–523.
10. Frey, S., and D. Görlich. 2009. FG/FxFG as well as GLFG repeats form a selective permeability barrier with self-healing properties. *EMBO J.* 28:2554–2567.
11. Milles, S., K. Huy Bui, ..., E. A. Lemke. 2013. Facilitated aggregation of FG nucleoporins under molecular crowding conditions. *EMBO Rep.* 14:178–183.
12. Yamada, J., J. L. Phillips, ..., M. F. Rexach. 2010. A bimodal distribution of two distinct categories of intrinsically disordered structures with separate functions in FG nucleoporins. *Mol. Cell. Proteomics.* 9:2205–2224.
13. Stewart, M. 2007. Molecular mechanism of the nuclear protein import cycle. *Nat. Rev. Mol. Cell Biol.* 8:195–208.
14. Chook, Y. M., and K. E. Süel. 2011. Nuclear import by karyopherin-βs: recognition and inhibition. *Biochim. Biophys. Acta.* 1813:1593–1606.
15. Bayliss, R., T. Littlewood, and M. Stewart. 2000. Structural basis for the interaction between FxFG nucleoporin repeats and importin-beta in nuclear trafficking. *Cell.* 102:99–108.
16. Bednenko, J., G. Cingolani, and L. Gerace. 2003. Importin beta contains a COOH-terminal nucleoporin binding region important for nuclear transport. *J. Cell Biol.* 162:391–401.
17. Isgro, T. A., and K. Schulten. 2005. Binding dynamics of isolated nucleoporin repeat regions to importin-beta. *Structure.* 13:1869–1879.
18. Mammen, M., S. K. Choi, and G. M. Whitesides. 1998. Polyvalent interactions in biological systems: implications for design and use of multivalent ligands and inhibitors. *Angew. Chem. Int. Ed.* 37:2755–2794.
19. Tu, L. C., G. Fu, ..., S. M. Musser. 2013. Large cargo transport by nuclear pores: implications for the spatial organization of FG-nucleoporins. *EMBO J.* 32:3220–3230.
20. Paradise, A., M. K. Levin, ..., J. H. Carson. 2007. Significant proportions of nuclear transport proteins with reduced intracellular mobilities resolved by fluorescence correlation spectroscopy. *J. Mol. Biol.* 365:50–65.
21. Kapinos, L. E., R. L. Schoch, ..., R. Y. H. Lim. 2014. Karyopherin-centric control of nuclear pores based on molecular occupancy and kinetic analysis of multivalent binding with FG nucleoporins. *Biophys. J.* 106:1751–1762.
22. Schoch, R. L., L. E. Kapinos, and R. Y. H. Lim. 2012. Nuclear transport receptor binding avidity triggers a self-healing collapse transition in FG-nucleoporin molecular brushes. *Proc. Natl. Acad. Sci. USA.* 109:16911–16916.
23. Schleicher, K. D., S. L. Dettmer, ..., R. Y. H. Lim. 2014. Selective transport control on molecular velcro made from intrinsically disordered proteins. *Nat. Nanotechnol.* 9:525–530.
24. Yang, W., and S. M. Musser. 2006. Nuclear import time and transport efficiency depend on importin beta concentration. *J. Cell Biol.* 174:951–961.

25. Uversky, V. N. 2013. Unusual biophysics of intrinsically disordered proteins. *Biochim. Biophys. Acta.* 1834:932–951.
26. Svitel, J., H. Boukari, …, P. Schuck. 2007. Probing the functional heterogeneity of surface binding sites by analysis of experimental binding traces and the effect of mass transport limitation. *Biophys. J.* 92:1742–1758.
27. Ribbeck, K., G. Lipowsky, …, D. Görlich. 1998. NTF2 mediates nuclear import of Ran. *EMBO J.* 17:6587–6598.
28. Bayliss, R., K. Ribbeck, …, M. Stewart. 1999. Interaction between NTF2 and xFxFG-containing nucleoporins is required to mediate nuclear import of RanGDP. *J. Mol. Biol.* 293:579–593.
29. Clarkson, W. D., H. M. Kent, and M. Stewart. 1996. Separate binding sites on nuclear transport factor 2 (NTF2) for GDP-Ran and the phenylalanine-rich repeat regions of nucleoporins p62 and Nsp1p. *J. Mol. Biol.* 263:517–524.
30. Clarkson, W. D., A. H. Corbett, …, M. Stewart. 1997. Nuclear protein import is decreased by engineered mutants of nuclear transport factor 2 (NTF2) that do not bind GDP-Ran. *J. Mol. Biol.* 272:716–730.
31. Stewart, M., H. M. Kent, and A. J. McCoy. 1998. The structure of the Q69L mutant of GDP-Ran shows a major conformational change in the switch II loop that accounts for its failure to bind nuclear transport factor 2 (NTF2). *J. Mol. Biol.* 284:1517–1527.
32. Bayliss, R., T. Littlewood, …, M. Stewart. 2002. GLFG and FxFG nucleoporins bind to overlapping sites on importin-beta. *J. Biol. Chem.* 277:50597–50606.
33. Schoch, R. L., and R. Y. H. Lim. 2013. Non-interacting molecules as innate structural probes in surface plasmon resonance. *Langmuir.* 29:4068–4076.
34. Bright, J. N., T. B. Woolf, and J. H. Hoh. 2001. Predicting properties of intrinsically unstructured proteins. *Prog. Biophys. Mol. Biol.* 76:131–173.
35. Miao, L., and K. Schulten. 2009. Transport-related structures and processes of the nuclear pore complex studied through molecular dynamics. *Structure.* 17:449–459.
36. Chaillan-Huntington, C., C. V. Braslavsky, …, M. Stewart. 2000. Dissecting the interactions between NTF2, RanGDP, and the nucleoporin XFXFG repeats. *J. Biol. Chem.* 275:5874–5879.
37. Bayliss, R., S. W. Leung, …, M. Stewart. 2002. Structural basis for the interaction between NTF2 and nucleoporin FxFG repeats. *EMBO J.* 21:2843–2853.
38. Morrison, J., J. C. Yang, …, D. Neuhaus. 2003. Solution NMR study of the interaction between NTF2 and nucleoporin FxFG repeats. *J. Mol. Biol.* 333:587–603.
39. Cushman, I., B. R. Bowman, …, M. S. Moore. 2004. Computational and biochemical identification of a nuclear pore complex binding site on the nuclear transport carrier NTF2. *J. Mol. Biol.* 344:303–310.
40. Isgro, T. A., and K. Schulten. 2007. Association of nuclear pore FG-repeat domains to NTF2 import and export complexes. *J. Mol. Biol.* 366:330–345.
41. Peleg, O., and R. Y. H. Lim. 2010. Converging on the function of intrinsically disordered nucleoporins in the nuclear pore complex. *Biol. Chem.* 391:719–730.
42. Opferman, M. G., R. D. Coalson, …, A. Zilman. 2012. Morphological control of grafted polymer films via attraction to small nanoparticle inclusions. *Phys. Rev. E Stat. Nonlin. Soft Matter Phys.* 86:031806.
43. Opferman, M. G., R. D. Coalson, …, A. Zilman. 2013. Morphology of polymer brushes infiltrated by attractive nanoinclusions of various sizes. *Langmuir.* 29:8584–8591.
44. Eisele, N. B., S. Frey, …, R. P. Richter. 2010. Ultrathin nucleoporin phenylalanine-glycine repeat films and their interaction with nuclear transport receptors. *EMBO Rep.* 11:366–372.
45. Cardarelli, F., L. Lanzano, and E. Gratton. 2012. Capturing directed molecular motion in the nuclear pore complex of live cells. *Proc. Natl. Acad. Sci. USA.* 109:9863–9868.
46. Tetenbaum-Novatt, J., L. E. Hough, …, M. P. Rout. 2012. Nucleocytoplasmic transport: a role for nonspecific competition in karyopherin-nucleoporin interactions. *Mol. Cell. Proteomics.* 11:31–46.
47. Peters, R. 2005. Translocation through the nuclear pore complex: selectivity and speed by reduction-of-dimensionality. *Traffic.* 6:421–427.
48. Dange, T., D. Grünwald, …, U. Kubitscheck. 2008. Autonomy and robustness of translocation through the nuclear pore complex: a single-molecule study. *J. Cell Biol.* 183:77–86.
49. Kubitscheck, U., D. Grünwald, …, R. Peters. 2005. Nuclear transport of single molecules: dwell times at the nuclear pore complex. *J. Cell Biol.* 168:233–243.
50. Macara, I. G. 2001. Transport into and out of the nucleus. *Microbiol. Mol. Biol. Rev.* 65:570–594.
51. Schreiber, G., and A. E. Keating. 2011. Protein binding specificity versus promiscuity. *Curr. Opin. Struct. Biol.* 21:50–61.
52. Terry, L. J., and S. R. Wente. 2007. Nuclear mRNA export requires specific FG nucleoporins for translocation through the nuclear pore complex. *J. Cell Biol.* 178:1121–1132.

Promiscuous Binding of Karyopherinβ1 Modulates FG Nucleoporin Barrier Function and Expedites NTF2 Transport Kinetics

Raphael S. Wagner[1], Larisa E. Kapinos[1], Neil J. Marshall[2], Murray Stewart[2] and Roderick Y. H. Lim[1*]

[1]Biozentrum and the Swiss Nanoscience Institute, University of Basel, Basel, Switzerland.

[2]MRC Laboratory of Molecular Biology, Cambridge, United Kingdom.

*Correspondence: roderick.lim@unibas.ch

Supporting Material

1. Sequence of FG domain fragments and purity of expressed proteins

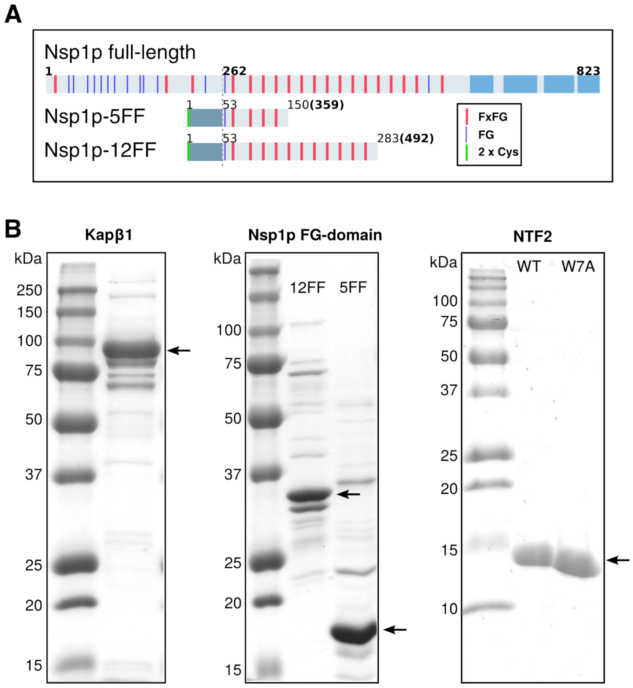

Figure S1. (A) Both FG domain fragments used in this study, Nsp1p-5FF and Nsp1p-12FF, contain regularly spaced FSFG repeats. Residue numbers in bold correspond to wild-type Nsp1p. The fragments contain a 52-residue 2xCys-/6xHis-/S-tag at their N-termini. The structured C-terminal domains of full-length Nsp1p are depicted in light blue. **(B)** 6% PAGE (0.1% SDS) of Kapβ1, 15% PAGE (0.1% SDS) of Nsp1p FG-domain fragments Nsp1p-12FF and Nsp1p-5FF, and 15% PAGE (0.1% SDS) of NTF2 and W7A-NTF2, respectively.

2. Regeneration of a Kapβ1 loaded Nsp1p-12FF layer

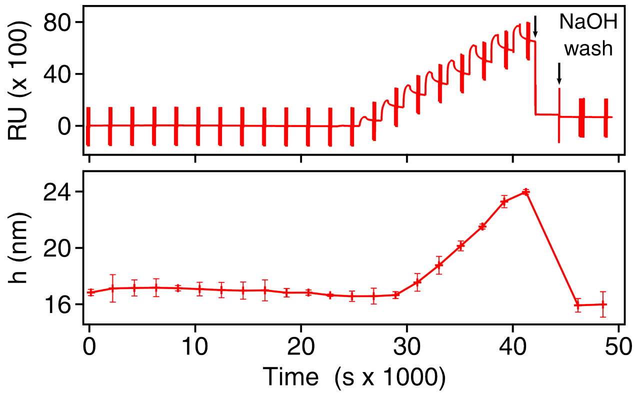

Figure S2. Nsp1p-12FF-bound Kapβ1 was removed after two short injections (30 s) of 0.2 M NaOH in PBS (pH ~13). This verifies that Kapβ1 is not covalently bound to the gold SPR sensor. Top: SPR response reaching baseline indicating Kapβ1 removal. Bottom: Corresponding Nsp1p-12FF layer height that is restored to its initial value after Kapβ1 removal.

3. Measuring FG domain layer height

1 % (w/v) non-interacting BSA molecules were injected and the corresponding SPR response (RU) was used to estimate a layer height, h, as described in detail by Schoch, *et al.* (1). The experimental parameters are summarized in Fig. S3.

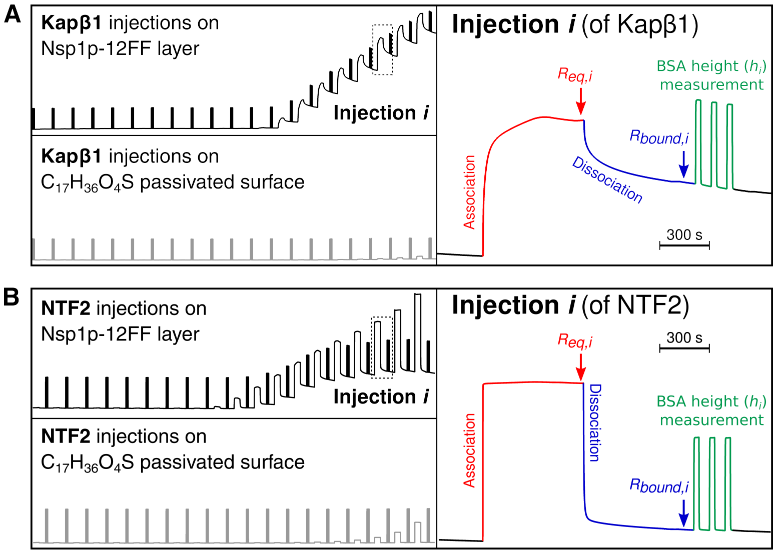

Figure S3. (A) Kapβ1 and **(B)** NTF2 were injected into Nsp1p functionalized and $C_{17}H_{36}O_4S$ passivated channels (left top and bottom, respectively). NTR-Nsp1p-12FF interactions consisted of an association (red) and dissociation (blue) phase followed by BSA injections (green). $R_{eq,i}$ corresponds to the SPR equilibrium binding response from each NTR injection of bulk concentration c_i with respect to the baseline (i.e., response at t = 0 s). $R_{bound,i}$ represents the SPR response for bound NTR after 480 s of dissociation (c_i = 0). Three consecutive BSA injections were used to measure the average layer height at layer occupancy $R_{bound,i}$. The BSA signal (green) returns to baseline indicating a lack of binding to Nsp1p FG domains, which is a prerequisite for reliable height measurements.

4. Multivalent binding kinetic analysis

The concept of surface heterogeneity as introduced by Svitel, *et al*. (2) was used to estimate the binding kinetics of Kapβ1 interacting with Nsp1p FG-domains as explained in detail by Kapinos *et al*. (3). The same approach was applied to NTF2 (and W7A-NTF2) binding using a simplified kinetic model assuming pseudo first-order kinetics:

$$L + D_S \underset{k_{off,\,i}}{\overset{k_{on,\,i}}{\rightleftharpoons}} LD_S \qquad \textbf{(S1)}$$

Here, L is the number of free binding sites, D_s is the NTF2 dimer concentration in solution and LD_s are binding sites occupied with dimeric NTF2 molecules.

Multivalent kinetic fits to representative SPR sensograms for NTF2, W7A-NTF2, Kapβ1 and NTF2 on Kapβ1 preloaded Nsp1p-12FF is given in Fig S4. The corresponding kinetic maps are shown in Fig. 4 and Fig. 6 in the main text.

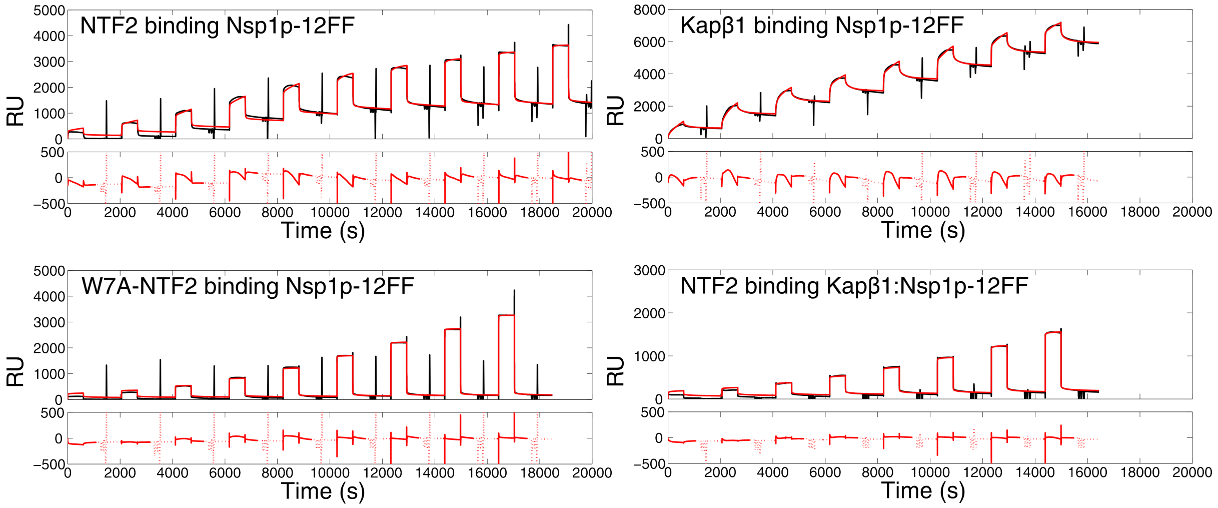

Figure S4. Representative SPR measurements (black), multivalent kinetic fits (red) and residuals shown for NTF2, W7A-NTF2, Kapβ1 and NTF2 binding Kapβ1 preloaded Nsp1p-12FF.

5. Dynamic Light Scattering

Hydrodynamic diameters where obtained by dynamic light scattering (DLS) in PBS with the addition of 1 mM DTT, using a Zetasizer Nano (Malvern). The measured hydrodynamic diameters were d_h = 8.7 ± 2.5 (polydispersity index = 0.3) for Nsp1p-5FF, d_h = 8.6 ± 2.7 (polydispersity index = 0.1) for Nsp1p-12FF, using n_p = 1.45 and n_d = 1.330 as the refractive index for proteins and dispersant, i.e., water; T = 298 K, viscosity = 0.8872 cP (1P = 0.1 Pa·s).

6. Estimation of Kapβ1 and NTF2 layers

The next-neighbor distance, g_{NTR}, between Kapβ1 or (W7A-) NTF2 molecules within the FG-domain layer was estimated as described (4). Accordingly, the number of layers is calculated using

$$Number\ of\ layers = \frac{d_h^2}{g_{NTR}^2} \qquad \textbf{(S2)}$$

where d_h is the respective NTR hydrodynamic diameter. SPR response units (RUs) are converted using the relation 1300 RU = 1 ng/mm^2 (1).

7. Equilibrium analysis of NTF2 binding to Nsp1p-5FF

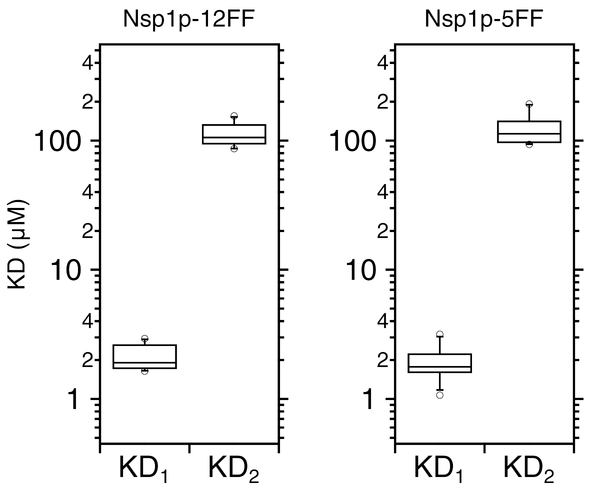

Figure S5. Equilibrium analysis shows that NTF2 binding is equivalent for both Nsp1p-5FF (N = 11) and Nsp1p-12FF (N = 15). The first and third quartiles are represented by box-plots. The median is given by the band inside the box. Whiskers represent the 9th and the 91th percentile. Outliers are plotted as open circles.

8. Multivalent kinetic analysis of NTF2 binding Nsp1p-5FF

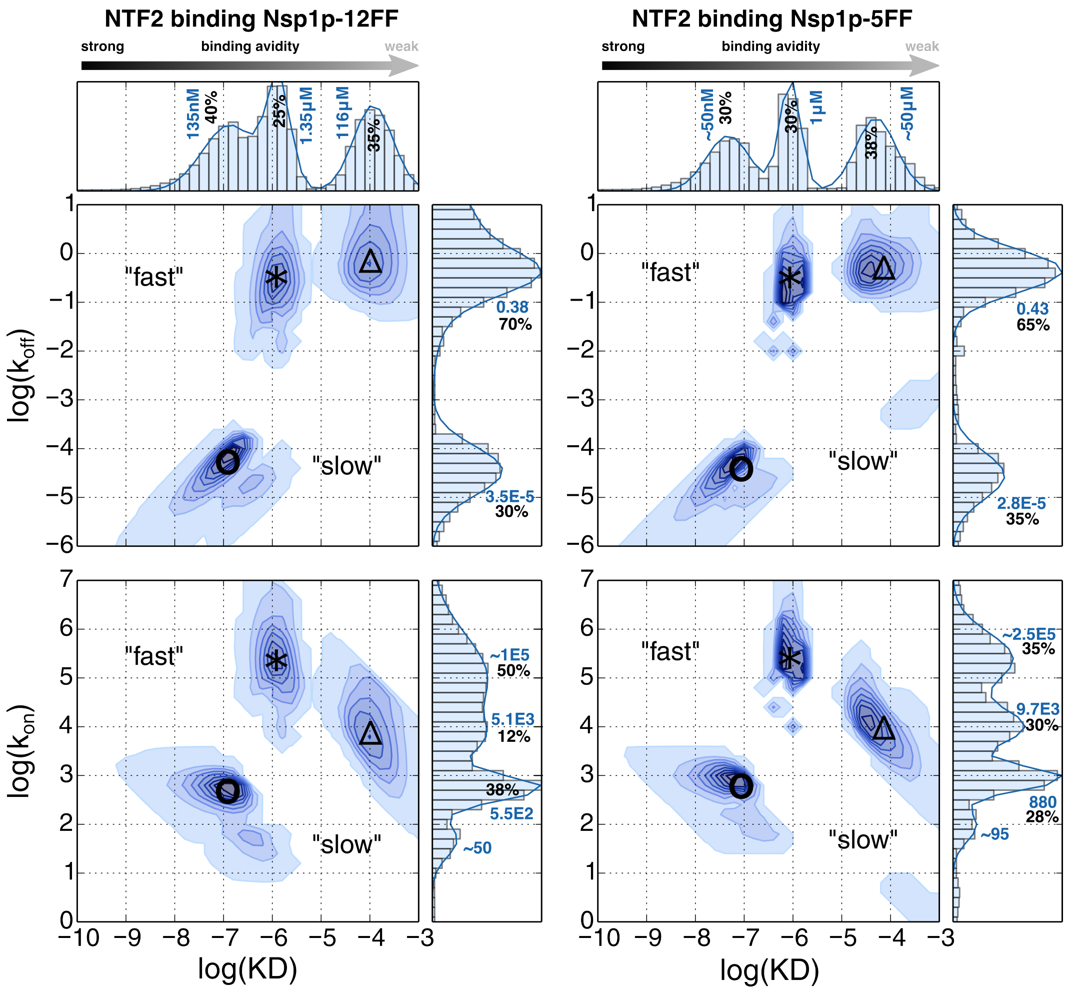

Figure S6. Multivalent binding analysis of NTF2 binding to close-packed layers of Nsp1p-12FF and Nsp1p-5FF. Two-dimensional interaction maps of kinetic on- and off-rates (k_{on}, k_{off}) are shown in relation to the equilibrium binding constant KD. The fractional abundance of different kinetic states is indicated by the color intensity and the sum over all values in a given axis is shown as accompanying histograms (top and right panels). Different kinetic species are labeled with o ("high-avidity slow-phase"), * ("mid-avidity fast-phase") and Δ ("low-avidity fast-phase"). Each distribution is given in percent of the total sum and their main values are depicted in bold. Units for k_{off} and k_{on} are s^{-1} and $s^{-1}M^{-1}$, respectively.

9. NTF2 binding to a Kapβ1-preloaded FG-domain layer

The total layer height did not change when comparing between the intrinsic dissociation of Kapβ1 in the presence and absence of NTF2 binding (Fig. S7).

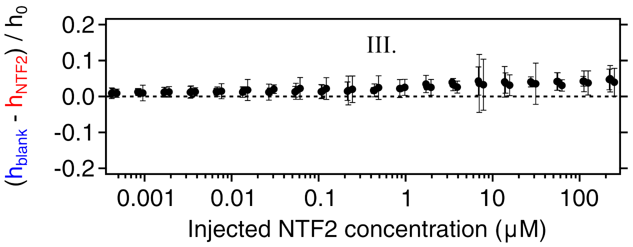

Figure S7. Similar height changes resulted from both NTF2 and blank injections on Kapβ1 preloaded Nsp1p-12FF brushes (normalized by the initial layer height h_0) as a function of NTF2 concentration.

To decouple the NTF2 from the Kapβ1 signal, the slow dissociation phase of Kapβ1 (see Fig. 5B and Fig. S8A) was fitted using a sum of exponential functions describing the dissociation of n = 3 different kinetic species forming binary complexes on the surface, modeled by

$$s(t) = \sum_n A_n e^{-k_{d,n} t} \quad \textbf{(S3)}$$

where $s(t)$ is the experimental SPR signal of Kapβ1, t is time, A_n the individual binding response at t = 0 of each species and $k_{d,n}$ is the apparent kinetic dissociation rate of species n. BSA injections were not included in the fit by using a mask in the fitting procedure.

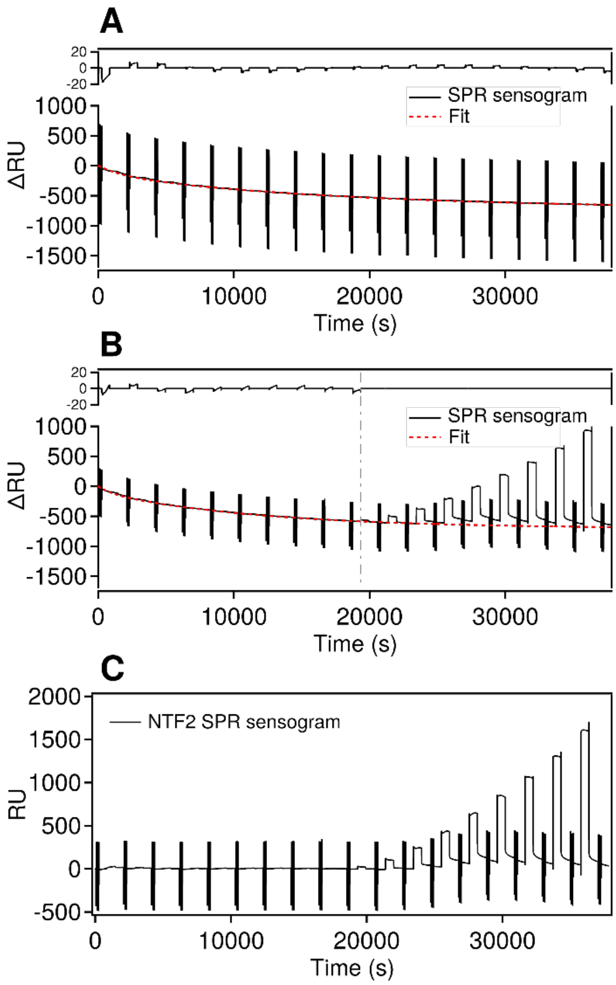

Figure S8. (A) Dissociation of Kapβ1 and fit with residual (top) in the absence of NTF2. **(B)** The dissociation of Kapβ1 was fitted for the first few injections (up to the dashed vertical line) where no significant change in SPR response was measured due to the low concentration of injected NTF2. Residual is shown on top. **(C)** NTF2 binding was isolated by subtracting the fit in (B) from the SPR sensogram of (B).

In a next step, the fit was applied to the dissociation phase of Kapβ1 in the presence of NTF2 binding (Fig. S8B). Finally, the fit obtained for the dissociation of Kapβ1 was subtracted from the total binding signal leaving only the SPR sensogram for NTF2 binding (Fig. S8C). This sensogram was then further analysed using the same protocol as described in Supporting Material 4.

10. Equilibrium analysis of NTF2 binding Kapβ1 preloaded Nsp1p-12FF

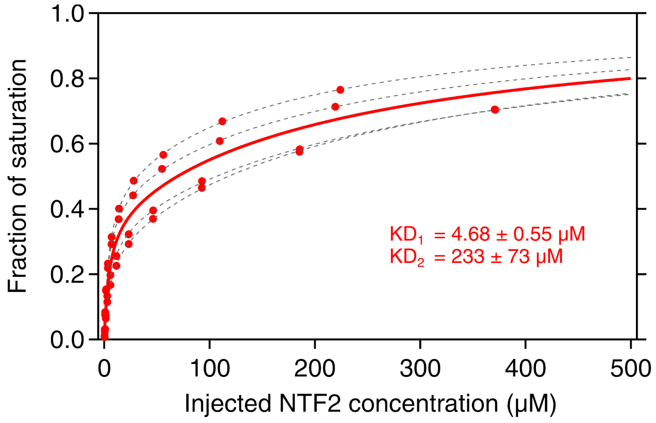

Figure S9. Equilibrium binding analysis of NTF2 binding Kapβ1 preloaded Nsp1p-12FF layers. The SPR equilibrium response was normalized by the maximum binding capacity (fraction of saturation) and shown as a function of injected bulk concentration. Grey dashed lines are individual 2-component Langmuir isotherm fits. The isotherm recalculated from the average KD_1 and KD_2 is shown as a solid line.

Supporting References

1. Schoch, R. L., and R. Y. H. Lim. 2013. Non-interacting molecules as innate structural probes in surface plasmon resonance. *Langmuir*. 29:4068-4076.
2. Svitel, J., H. Boukari, D. Van Ryk, R. C. Willson, and P. Schuck. 2007. Probing the functional heterogeneity of surface binding sites by analysis of experimental binding traces and the effect of mass transport limitation. *Biophys. J.* 92:1742-1758.
3. Kapinos, L. E., R. L. Schoch, R. S. Wagner, K. D. Schleicher, and R. Y. H. Lim. 2014. Karyopherin-centric control of nuclear pores based on molecular occupancy and kinetic analysis of multivalent binding with FG nucleoporins. *Biophys. J*. 106:1751-1762.
4. Schoch, R. L., L. E. Kapinos, and R. Y. H. Lim. 2012. Nuclear transport receptor binding avidity triggers a self-healing collapse transition in FG-nucleoporin molecular brushes. *Proc. Natl. Acad. Sci. USA*. 109:16911-16916.
